# Alterations of specific chromatin conformation affect ATRA-induced leukemia cell differentiation

**DOI:** 10.1038/s41419-017-0173-6

**Published:** 2018-02-08

**Authors:** Yanjian Li, Yi He, Zhengyu Liang, Yang Wang, Fengling Chen, Mohamed Nadhir Djekidel, Guipeng Li, Xu Zhang, Shuqin Xiang, Zejun Wang, Juntao Gao, Michael Q. Zhang, Yang Chen

**Affiliations:** 1 MOE Key Laboratory of Bioinformatics, Bioinformatics Division and Center for Synthetic & Systems Biology, TNLIST, School of Medicine, Tsinghua University, Beijing, 100084 China; 2 MOE Key Laboratory of Bioinformatics, Bioinformatics Division and Center for Synthetic & Systems Biology, TNLIST, Department of Automation, Tsinghua University, Beijing, 100084 China; 3 Department of Biological Sciences, Center for Systems Biology, The University of Texas, Dallas 800 West Campbell Road, RL11, Richardson, TX 75080-3021 USA; 4 School of Pharmaceutical Sciences, Tsinghua University, Beijing, 100084 China

## Abstract

Chromatin conformation plays a key role in regulating gene expression and controlling cell differentiation. However, the whole-genome chromatin conformation changes that occur during leukemia cell differentiation are poorly understood. Here, we characterized the changes in chromatin conformation, histone states, chromatin accessibility, and gene expression using an all-trans retinoic acid (ATRA)-induced HL-60 cell differentiation model. The results showed that the boundaries of topological associated domains (TADs) were stable during differentiation; however, the chromatin conformations within several specific TADs were obviously changed. By combining H3K4me3, H3K27ac, and Hi-C signals, we annotated the differential gene-regulatory chromatin interactions upon ATRA induction. The gains and losses of the gene-regulatory chromatin interactions are significantly correlated with gene expression and chromatin accessibility. Finally, we found that the loss of GATA2 expression and DNA binding are crucial for the differentiation process, and changes in the chromatin structure around the *GATA2* regulate its expression upon ATRA induction. This study provided both statistical insights and experimental details regarding the relationship between chromatin conformation changes and transcription regulation during leukemia cell differentiation, and the results suggested that the chromatin conformation is a new type of potential drug target for cancer therapy.

## Introduction

Hierarchical conformations formed by chromosomes play a pivotal role in the regulation of gene expression. Previous studies have uncovered that individual chromosomes occupy specific territories^1–3^, and gene transcription has been shown to be correlated with the gene’s nuclear position relative to the nuclear lamina and the bulk of the chromosome territories^4,5^. Recent studies using high-throughput chromosome conformation capture (Hi-C) have revealed that genomes are organized into topological associated domains (TADs) of several hundred kilo-bases to one mega-base in length, and chromatin regions in a TAD are more likely to interact with other regions within the same TAD than with regions outside of the TAD. Most TAD locations have been shown to be invariant across cell types^6,7^ and appeared to be evolutionarily conserved^8,9^. However, genes within the same TADs reflect coordinated changes in expression upon hormone stimulation or during differentiation^10–14^, indicating that TADs act not only as structure units but also as function units for transcription regulation. Furthermore, within the TADs, long-range chromatin interactions mediated by specific proteins or noncoding RNAs connect distal regulatory regions, such as enhancers and gene promoters, enabling the long-range regulation of gene expression^15–17^.

In leukemia, chromatin conformation has been shown to play an important role in transcription regulation and drug response^18,19^. The dynamic chromatin conformation signatures in the HOXA locus have been shown to correlate with gene expression dynamics and are used to classify the leukemia types^20,21^. In addition, studies have demonstrated that the major regulator of leukemia cell differentiation, i.e., c-Myb, could be modulated by upstream regulatory regions through chromatin interaction^22^. At the whole genome level, the global changes in chromatin conformation caused by cohesin mutations have been shown to inhibit normal differentiation and induce leukemia^23,24^, emphasizing the importance of chromatin conformation in leukemogenesis and leukemia therapies. However, the whole-genome chromatin conformation change during leukemia cell differentiation and its relationship with transcription regulation have not been well characterized.

To address this issue, all-trans retinoic acid (ATRA)-induced HL-60 cells were used in this study as a well-known model of leukemia cell differentiation^25–27^. We performed chromosome conformation capture followed by high-throughput sequencing (Hi-C), chromatin immunoprecipitation combined with high-throughput sequencing (ChIP-seq), Transposase-Accessible Chromatin with high-throughput sequencing (ATAC-seq), and RNA sequencing (RNA-seq) analyses and observed a non-random distribution of differentially expressed genes (DEGs) among the TADs. Within the TADs, the chromatin interactions between genes and regulatory regions were shown to be positively correlated with the gene expression level and chromatin accessibility. Both the expression and DNA binding of the key transcription factor GATA2 were downregulated upon ATRA induction; meanwhile, the chromatin interactions between the GATA2 promoter and upstream enhancers were lost, accompanied by chromatin accessibility changes in the enhancers.

## Results

### Compartment and TAD position did not significantly change upon ATRA induction

To determine whether the whole-genome chromatin conformation changes during ATRA-induced differentiation, we generated Hi-C libraries from 2 independent biological replicates of control and ATRA-treated HL-60 cells (Fig. S1) using a modified in situ Hi-C protocol (Methods). The reproducibility between the biological replicates was high (Fig. S4). To obtain a higher resolution, we combined the data from the replicates to obtain a total of ~160 million (ATRA-treated) and ~150 million (control) interactions for the subsequent analysis.

**Fig. 1:**
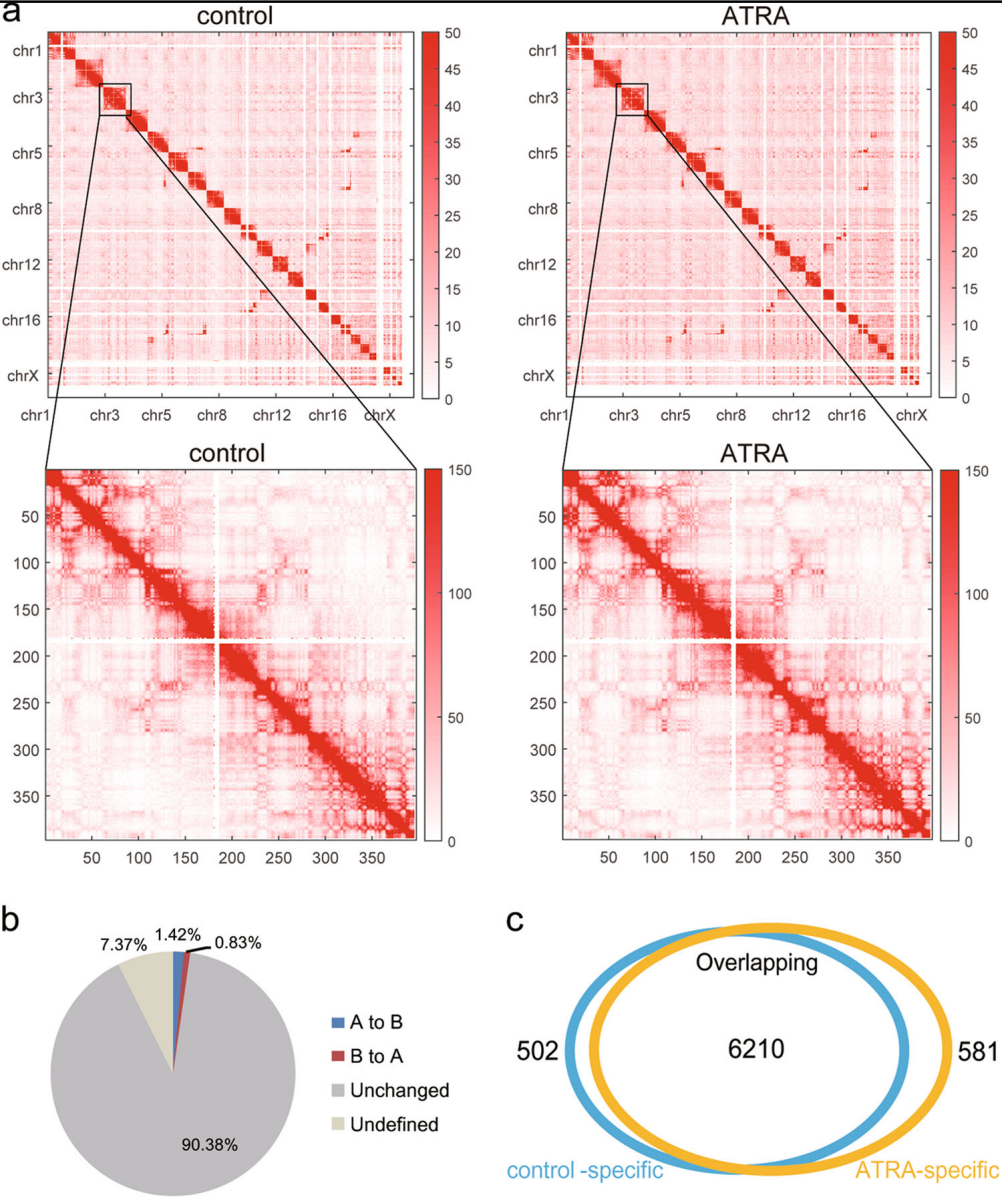
Chromatin conformation landscapes during ATRA-induced HL-60 differentiation **a** (upper panels) Whole-genome Hi-C heatmaps using 1-Mb bin of control (left) and ATRA-treated (right) HL-60 cells. Chromosomes are stacked from top left to bottom right in order (from chr1 to chrX). (lower panels) Enlarged intra-chromosomal interaction heatmaps of chr3. Color axis is shown on the right. **b** Pie chart showing the compartment changes between the control and ATRA-treated HL-60 cells. “Unchanged” indicates that the compartments are identical in both cells. “A to B” indicates that the compartments have an A pattern in the control cells and a B pattern in the ATRA-treated cells. “B to A” indicates that the compartments have a B pattern in the control cells and an A pattern in the ATRA-treated cells. “Undefined” indicates regions in which the compartment pattern could not be defined (projection of Hi-C first principal component equals zero). **c** Venn diagram showing that more than 90% of the TAD boundaries are stable in the control and ATRA-treated cells

By visualizing the data in a heatmap, the Hi-C data revealed a hierarchical chromatin conformation and self-interacting pattern in the HL-60 cells (Fig. S5). While the prominent lobulation of the cell nucleus could be observed in the ATRA-treated cells under a fluorescence microscope (Fig. S3)^27^, the contact frequency between each chromosome appeared unchanged in the Hi-C data (Fig. 1a). Within a chromosome, we observed a similar organization pattern along the heatmap diagonal in the both the control and ATRA-treated cells (Fig. S5), suggesting that the basic structural units of the genome remained stable upon the ATRA induction.

To determine whether the subscale chromatin conformations changed upon the ATRA induction, we performed a compartment classification in the control and ATRA-treated cells and compared the results. Upon the ATRA induction, only 0.81% of the genome exhibited a changed compartment pattern from A to B, and 0.48% of the genome exhibited a changed compartment pattern from B to A (Fig. 1b, S6), implying that the genome compartmentalization was generally conserved.

TADs have been shown to act as both structural and functional units in the genome^9,14,28^. To further describe the TAD variation with or without the ATRA treatment, we identified the TADs using the clustering-based Hi-C domain finder (CHDF) method^29^. In total, 5908 and 5774 TADs with an average length of ~400 kb were identified in the control and ATRA-treated cells, respectively (Fig. S7). Integrating with the ENCODE HL-60 ChIP-seq data, we observed an enrichment of CTCF at the TAD boundaries (Fig. S7). A strong enrichment of GAPBA was also detected (Fig. S8), which was similar to that observed in MCF-7 cells^30^. Consistently with previous chromatin conformation studies^10^, ~90% of the TAD boundaries detected in the control data remained similarly positioned following the ATRA treatment (Fig. 1c). Furthermore, up to 95% of the boundaries moved within 200 kb, and nearly half of the specific boundaries were located in regions without any expressed genes. Thus, we concluded that the chromatin conformation and TAD location did not substantially change at the compartment level after the ATRA-induced differentiation.

### TADs with significant internal chromatin interaction changes enrich differentially expressed genes

**Fig. 2:**
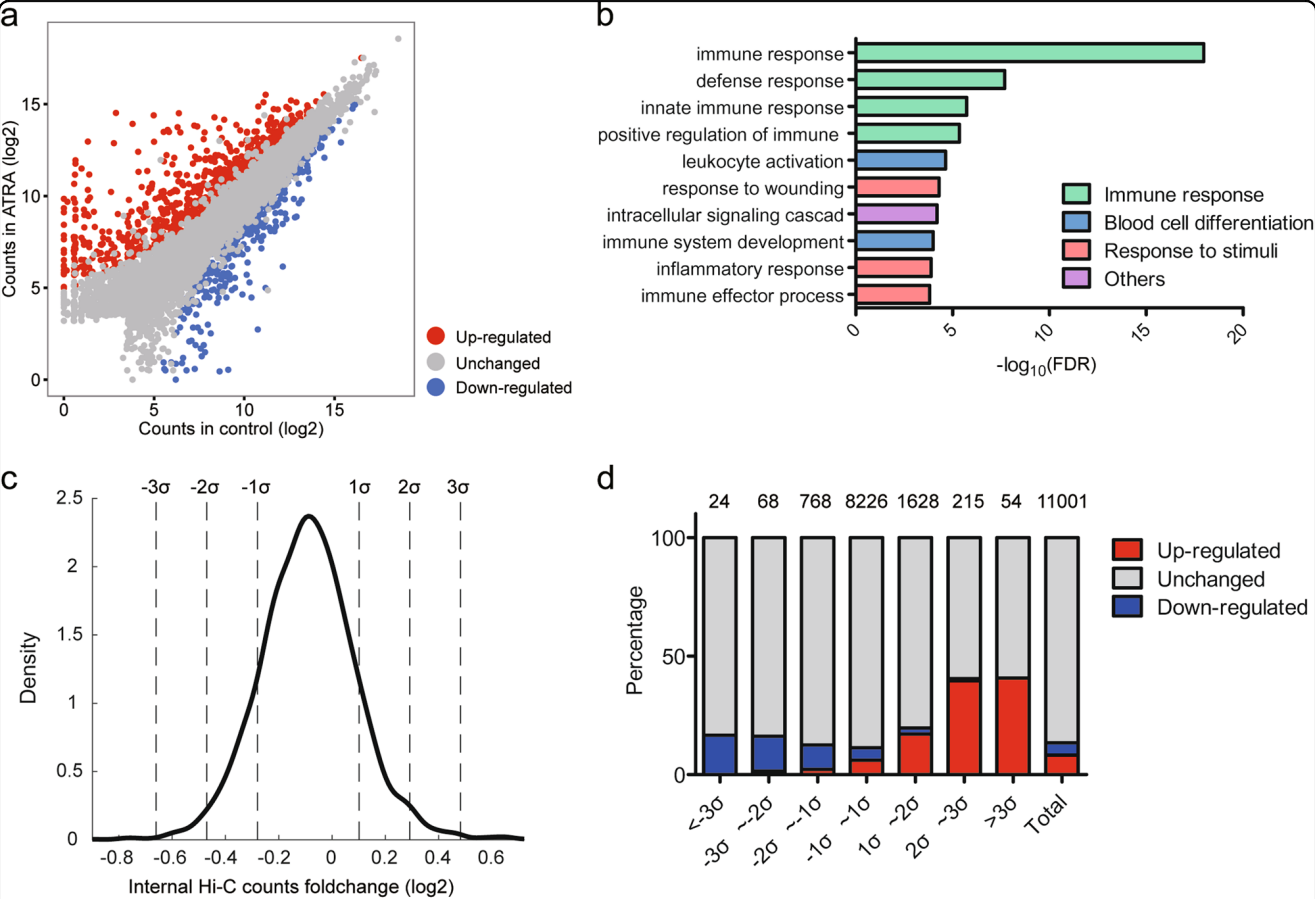
Distribution of the differentially expressed genes in TADs **a** Scatter plot of the differential gene expression between the control and ATRA-treated HL-60 cells. Red dots denote genes with significantly upregulated expression in the ATRA-treated cells (log2(foldchange)>0.9, adjusted *p*-value < 0.01). Blue dots denote genes with significantly downregulated expression in the ATRA-treated cells (log2(foldchange)<−0.9, adjusted *p*-value < 0.01). Gray dots denote genes with no significant expression change. **b** Top 10 GO terms of differentially expressed genes between the control and ATRA-treated HL-60 cells. FDR false discovery rate. **c** Probability density of TADs with at least one gene expression change based on the internal Hi-C counts. *σ* represents the standard deviation of all TAD log2 internal Hi-C counts. **d** Accumulation bar-plot showing the enrichment of genes in TADs. The TADs are divided into seven categories as shown in Fig. 2c, and total represents all expressed genes located in TADs. Number of genes located in each type of TAD is shown above the bar

To investigate the underlying functional changes in gene expression upon ATRA induction, we performed RNA-seq in two replicates of control and ATRA-treated cells (Fig. S9). The DEGs analysis revealed 941 upregulated and 611 downregulated genes following the ATRA induction (Fig. 2a). A Gene Ontology (GO) analysis of biological process showed that the DEGs were significantly enriched in “immune response” and “leukocyte activation”, which is consistent with the terminal differentiation process of neutrophils (Fig. 2b, S10).

Then, we determined whether the gene expression changes correlated with the chromatin interaction changes within the TADs. Thus, we ranked the TADs containing the expressed genes (*n* = 3362) according to the internal Hi-C count fold-change and divided these TADs according to their deviation from the average fold change of all TADs and calculated the distribution of the expressed genes in these TADs (Fig. 2c). The results showed that the genes in TADs with distinct interaction changes were more likely to be differentially expressed following the ATRA induction than those in TADs with moderate interaction changes. Furthermore, the TADs with increased chromatin interactions enriched the upregulated genes while the TADs with decreased chromatin interactions showed the opposite result (Fig. 2d). Thus, our data revealed the presence of a positive correlation between gene expression and the internal chromatin interaction frequency within the TADs.

The chromatin conformation and transcription changes have been reported to correlate with the histone modification states within TADs^31^. To characterize the epigenetic states within the TADs, we performed ChIP-seq in the control and ATRA-treated cells using antibodies against H3K4me3 and H3K27ac that mainly mark the active promoters and enhancers, respectively. By calculating the ChIP-seq signal changes within the TADs, the TADs with increased chromatin interactions showed relatively increased levels of H3K4me3 and H3K27ac, whereas the TADs with decreased chromatin interactions showed an opposite change (Fig. S11). This finding is consistent with the model in which the alteration of epigenetic activity within a TAD may have a positive impact on the chromatin conformation variation and the differential gene expression, corroborating a previous study in a short-time hormone induction model^10^.

### Gene-regulatory chromatin interactions were altered upon ATRA induction

**Fig. 3:**
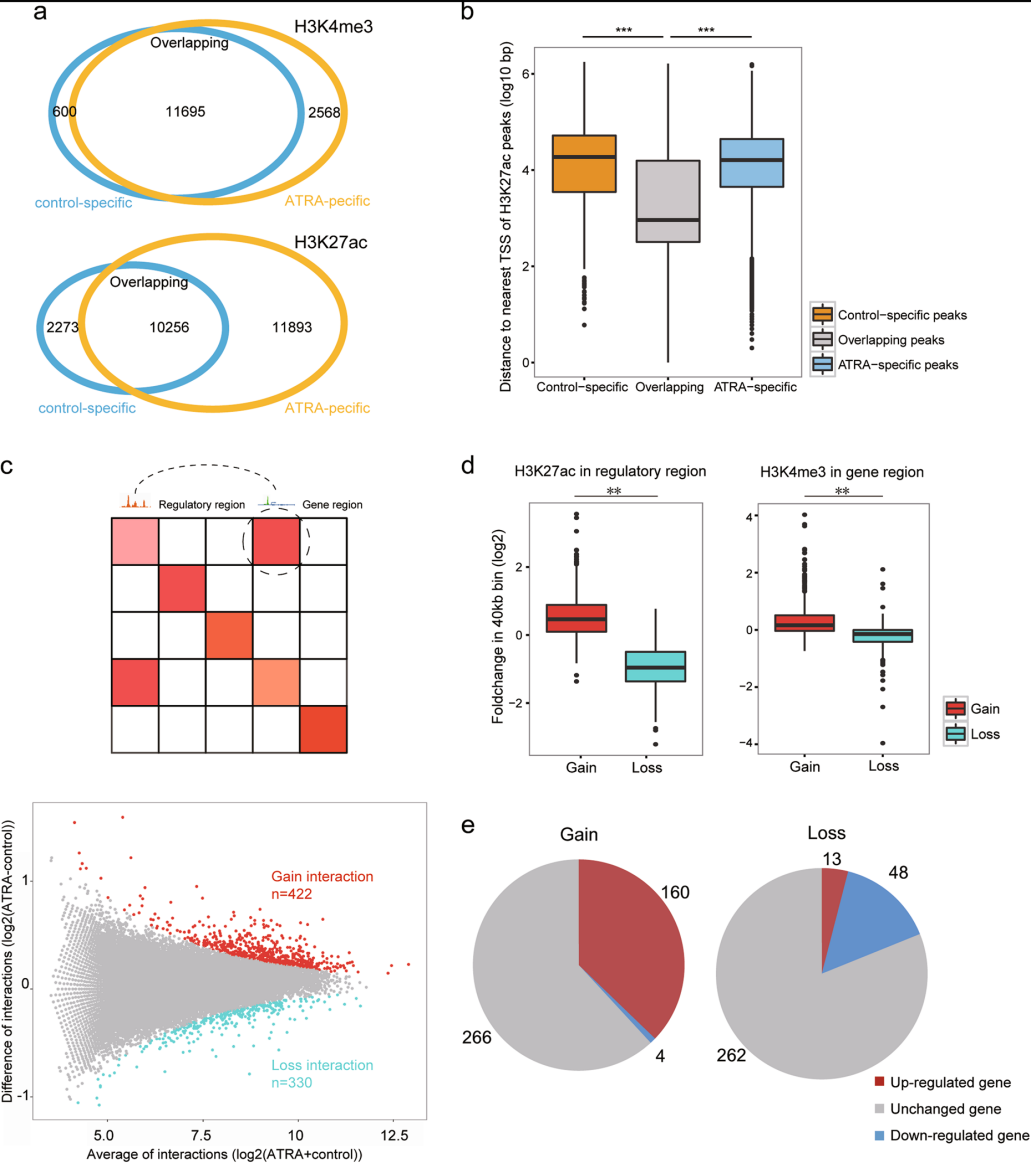
Differential gene-regulatory interaction analysis upon ATRA induction **a** Venn diagram showing the H3K4me3 peak overlaps between the control and ATRA-treated HL-60 cells (upper panel). Similar diagram showing the H3K27ac peak overlaps (lower panel). **b** Boxplot showing the log10 distance to the nearest TSS of the overlapping ATRA-specific and control-specific H3K27ac peaks shown in Fig. 3a. Specific peaks are significantly farther from the TSS than overlapping peaks. The *p*-value was determined by a Mann–Whitney *U*-test: **p-value < 0.001, ***p-value < 0.0001. **c** (upper panel) Schematic diagram of the gene-regulatory interaction definition. H3K27ac peaks distal to the TSS and H3K4me3 peaks in the promoters are used to define the regulatory regions and gene regions, respectively. Hi-C counts in corresponding bin represent the gene-regulatory interaction intensity. (lower panel) MA-plot showing the differential interaction analysis. Red dots denote interactions with enhanced intensities in the ATRA-treated cells (referred to as Gain, *p*-value with Benjamini correction < 0.001). Cyan dots denote interactions with weakened intensities in the ATRA-treated cells (referred to as Loss, *p*-value with Benjamini correction < 0.001). **d** Boxplot showing that the H3K27ac signal in regulatory regions (left) and H3K4me3 signal in gene regions (right) are increased in Gain interactions and decreased in Loss interactions upon ATRA induction. The *p*-value was determined by Mann–Whitney *U*-test: **p-value < 0.001, ***p-value < 0.0001. **e** Pie chart showing that gain interactions enrich more upregulated genes and loss interactions enrich more downregulated genes

To annotate the active promoters and enhancers during the differentiation process, we used the ChIP-seq data and identified 12,295 H3K4me3 peaks and 1,2493 H3K27ac peaks (Methods) in the ATRA-treated cells (14,263 H3K4me3 and 22,149 H3K27ac peaks were identified in the control, Fig. 3a). By calculating the distance to the nearest TSS of each H3K27ac peak, we found that the ATRA and control-specific peaks were frequently located farther from the TSS than the common peaks (Fig. 3b), indicating the occurrence of more distal regulatory changes following the ATRA induction.

Chromatin interactions can move distal regulatory elements, promoters and the TSS to the proximity required for transcription regulation^32,33^. To describe these functional chromatin interactions, we first divided the whole genome into 40 kb bins. Next, the bin was annotated as a “gene region” if it contained the promoters of the expressed genes or a “regulatory region” if it contained H3K27ac peaks distal to the promoter (Fig. 3c). Then, the Hi-C contacts between a gene region and a regulatory region were used to represent the gene-regulatory interaction intensity. We selected differential gene-regulatory interactions according to the significance of the difference in the Hi-C contacts between the control and ATRA-treated cells (*p*-value with Benjamini correction <0.001, see Methods) and identified 422 pairs of increased-intensity gene-regulatory interactions (referred to as “Gain”) and 330 pairs of reduced-intensity interactions (referred to as “Loss”) (Fig. 3c). The changes in the gene-regulatory interactions were coordinated with the whole TAD chromatin interaction alterations (Fig. S12). Comparing the fold changes in the H3K27ac signal in the regulatory bins and the H3K4me3 signals in the corresponding gene regions following the ATRA induction, we found that both signals were significantly increased in Gain interactions, while those in Loss interactions showed the opposite trend (Fig. 3d). These results can, at least partially, explain the positive correlation between active histone states and chromatin interaction intensities and the corresponding positive correlation with the differential gene expression that was initially observed. Indeed, 430 and 323 genes were involved in the Gain and Loss gene-regulatory chromatin interactions, respectively; of these genes, 164 and 61 were differentially expressed upon the ATRA induction (Fig. 3e). The proportion of DEGs involved in the differential gene-regulatory chromatin interactions was significantly (Fisher’s exact test *p*-value < 1×e^−22^) higher than that in the whole genome (12,266 total genes with 1649 DEGs). Thus, the Gain or Loss interactions likely caused the corresponding genes to upregulate or downregulate, respectively. This finding indicates that most gene-regulatory interactions likely enhance or at least facilitate the target gene expression, which may be in stark contrast to the majority of intergenic interactions in the heterochromatin regions that repress or silence gene expression^32,34^.

### Differential gene-regulatory chromatin interactions were correlated with the changes in chromatin accessibility

**Fig. 4:**
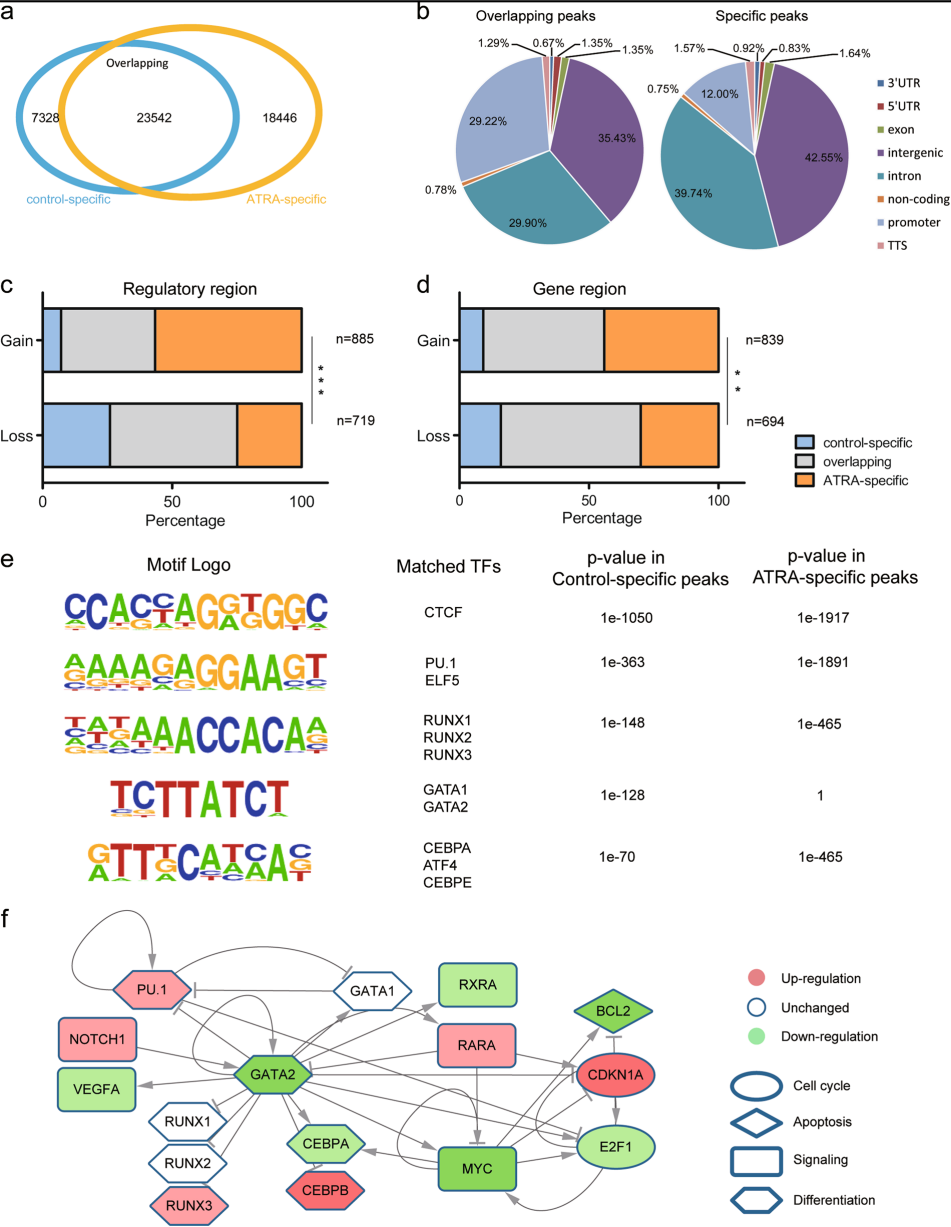
Changes in chromatin accessibility correlated with gene-regulatory interaction variation **a** Venn diagram showing the ATAC-seq peak overlaps between the control and ATRA-treated HL-60 cells. **b** Pie chart showing the annotation of the overlapping and specific ATAC-seq peaks. Proportion of promoter peaks is significantly lower in the specific peaks than that in the overlapping peaks. **c** Accumulation bar-plot showing the enrichment of ATAC-seq peaks in regulatory regions of Gain or Loss interactions. *p*-Value was determined by Fisher exact test: **p-value < 1e−10, ***p-value < 1e−22. **d** Similar to Fig. 4c showing enrichment in gene regions. **e** Homer de novo motif analysis of specific ATAC-seq peaks. Motifs are ordered by significance in the control-specific peaks from top to bottom. GATA motif was only significantly enriched in the control-specific peaks. **f** Core transcription regulation network of the ATRA-induced differentiation process. Shapes of nodes represent their biological function, including cell cycle (ellipse), apoptosis (diamond), signaling (rectangle), and differentiation (hexagon). Colors of nodes represent expression changes upon ATRA induction, including upregulation (red), downregulation (green), and unchanged (white)

The binding of transcription factors on accessible chromatin regions regulates enhancer activity and gene expression and orchestrates cell differentiation^35,36^. To characterize the chromatin accessibility changes upon the ATRA induction, we performed ATAC-seq in independent biological replicates of control and ATRA-treated cells. We identified 41,988 peaks in the ATRA-treated cells and 30,870 peaks in the control cells. Overall, 18,446 peaks could only be identified in the ATRA-treated cells (referred as ATRA-specific peaks), while 7328 peaks could only be identified in the control cells (referred as control-specific) (Fig. 4a). By annotating the ATAC-seq peaks according to their genome location, we noticed that the proportion of promoters in the specific peaks was lower than that in the overlapped peaks (Fig. 4b), suggesting that the chromatin accessibility changes occurred mainly in distal regulatory regions.

To uncover the correlation between the differential gene-regulatory chromatin interactions and transcription factor binding. We counted the ATAC-seq peaks located in the gene and regulatory regions of the differential interactions and found that Gain and Loss interactions enriched the ATRA-specific and control-specific peaks, respectively (Fig. 4c). The regulatory regions showed a stronger enrichment tendency than the gene regions, suggesting that the changes in TF binding in the open chromatin regions, particularly in distal regulatory regions, modulate the formation of the chromatin interactions.

Next, to characterize the transcription factor binding states following ATRA induction, we performed a motif analysis in control specific or ATRA specific ATAC-seq peaks using the software HOMER. Most transcription factors, including CTCF, PU.1, RUNX, and CEBP, were highly similar between the ATRA-treated or control cells (Fig. 4d). A modest upregulation of PU.1 mRNA (~1.9-fold) was observed, indicating that PU.1 was activated by ATRA and bound to open chromatin to orchestrate the differentiation process. Among the RUNX family members, only RUNX3 showed significant mRNA level changes following the ATRA treatment (~3.2-fold, upregulated), suggesting that it potentially performs a regulatory function in ATRA induction. In addition, we observed that both binding motifs and the expression of GATA2 were enriched only in the control cells (Fig. 4d, S14, S16). GATA2 mRNA was significantly downregulated after differentiation (~0.06-fold, downregulated), suggesting that the loss of GATA2 binding may facilitate the ATRA induction process. To further delineate the relationship between the transcription factors and DEGs during differentiation, we mapped the above-mentioned transcription factors and DEGs to the HTRI TF-Target network (Fig. S15)^37^. In the network, we found that GATA2 acted as the most important hub node with the highest network degree. Furthermore, GATA2 exhibited interactions with most transcription factors enriched with ATAC-seq peaks and known key regulatory factors for granulocytes differentiation (Fig. 4f). Altogether, by integrating chromatin accessibility information and the transcription regulatory network, we showed that GATA may act as a crucial transcription factor during ATRA-induced HL-60 differentiation.

### ATRA induction decreased the chromatin interactions between the GATA2 promoter and regulatory regions

**Fig. 5:**
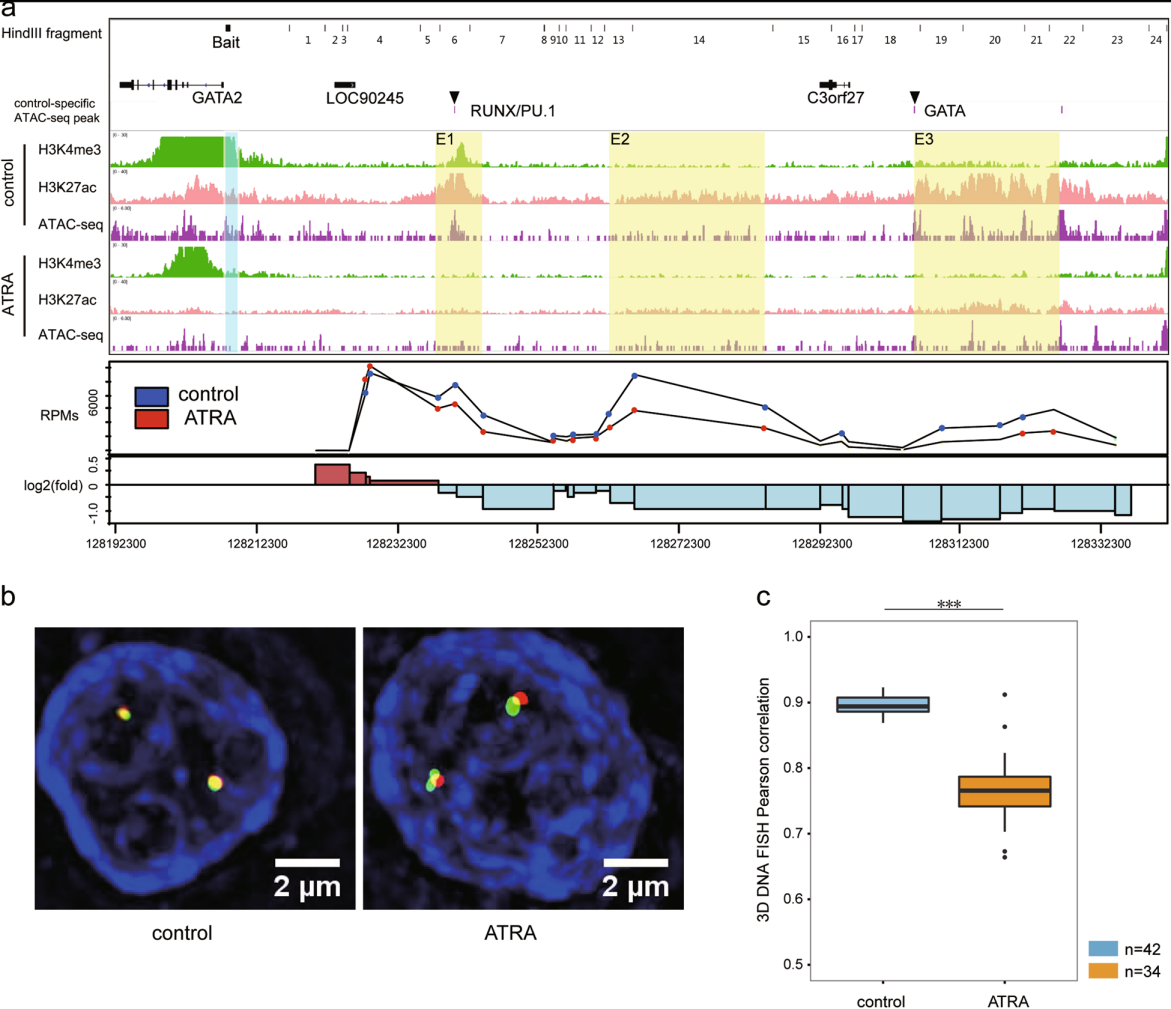
Chromatin conformation in GATA2 locus is significantly changed upon ATRA induction **a** Diagram showing ChIP-seq, ATAC-seq, and 4C-seq results of GATA2 promoter and upstream regions. (upper panel) HindIII cutting sites, gene annotation, and control-specific ATAC-seq peaks. Transcription factors with enriched motifs in the peak are labeled. (middle panel) Genome browser views of ChIP-seq and ATAC-seq results of control and ATRA-treated HL-60 cells; semitransparent cyan color labels the bait site used in 4C-seq, and semitransparent yellow color labels the putative enhancers recognized in 4C-seq. (lower panel) 4C-seq results of control and ATRA-treated HL-60 cells using the bait mentioned above. Red dots indicate 4C-seq RPMs in the ATRA-treated cells, and blue dots indicate RPMs in the control cells; only fragments with significant interactions with the bait are shown. Log2-foldchange of RPMs is shown in the lowest bar graph. **b** DNA FISH showing the loss of the chromatin loop between the GATA2 promoter and enhancer. BAC probes containing the promoter (red) and enhancer (green) were more separated in the ATRA-treated cells (right) than in the control cells (left). **c** Loci of promoter and enhancer interacted more in the control cells than in the ATRA-treated cells. *p*-Value was determined by Mann–Whitney *U*-test: **p-value < 0.001, ***p-value < 0.0001

According to the differential gene-regulatory chromatin interaction analysis, we observed a significant decrease in the interactions between gene regions containing *Gata2* and upstream regulatory regions following the ATRA stimulation (Fig. S16). To characterize the chromatin conformation relating to *Gata2* in detail, we performed circular chromatin conformation capture (4C) in the control and ATRA-treated cells using the *Gata2* promoter as bait. In the control cells, we found that the promoter of *Gata2* had strong chromatin interactions primarily with three upstream enhancers (i.e., chr3:128240590–128254410, 128262419–128292429, and 128309790–128334446) marked by H3K27ac peaks. The position of enhancer E3 (~80 kb upstream of the GATA2 promoter) was very close to a known enhancer^38^, confirming the reliability of our 4C data. After the ATRA stimulation, the interaction intensities between the *Gata2* promoter and upstream regions decreased in varying degrees (0.54-, 0.46-, and 0.4-fold for E1, E2, and E3, respectively) consistently with the decrease in H3K27ac (Fig. 5a). The motif analysis showed that these regions harbored the PU.1/RUNX1 and GATA motif separately, and the open chromatin state was only observed in the control cells (Fig. 5a). The loss of key transcription factor (PU.1/RUNX1/GATA) binding at distal regulatory regions appears to break the chromatin loop and suppress GATA2 expression. To further validate the loop disappearance upon ATRA induction, we performed three-dimensional DNA fluorescent in situ hybridization (FISH) in the control and ATRA-treated cells. The probes representing the *Gata2* promoter and enhancer E3 showed a greater overlap in the control cells than in the ATRA-treated cells (Fig. 5b). Additionally, the Pearson correlation coefficient between the two probes was higher in the control cells than that in the ATRA-treated cells, further verifying the broken loop upon ATRA induction (Fig. 5c).

### Loss of 5′–3′ loop may inhibit ZBTB16 mRNA expression

**Fig. 6:**
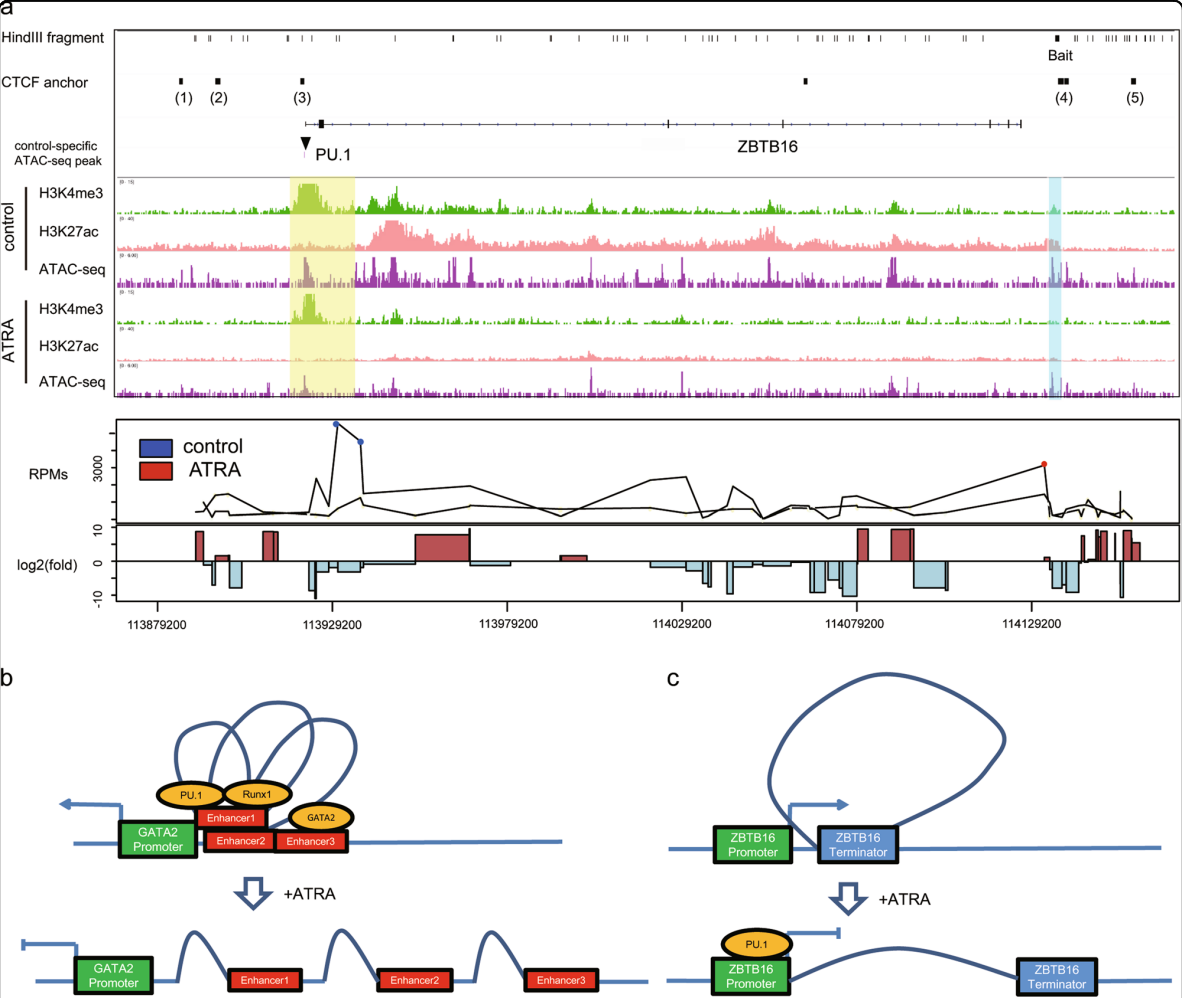
5′–3′ loop of ZBTB16 was lost upon ATRA induction **a** Diagram showing ChIP-seq, ATAC-seq and 4C-seq results of ZBTB16. (upper panel) HindIII cutting sites, gene annotation and control-specific ATAC-seq peaks. Transcription factors with enriched motifs in the peak are labeled. (middle panel) Genome browser views of ChIP-seq and ATAC-seq results of ATRA-treated and control HL-60 cells; semitransparent cyan color labels the bait site used in 4C-seq, and semitransparent yellow color labels the site with a strong interaction with the bait recognized in 4C-seq. (lower panel) 4C-seq results of ATRA-treated and control HL-60 cells using the bait mentioned above. Red dots indicate 4C-seq RPMs in the ATRA-treated cells, and blue dots indicate RPMs in the control cells; only fragments with significant interactions with the bait are shown. Log2-foldchange of RPMs are shown in the lowest bar graph. **b** Model depicting the change in the chromatin architecture around the *Gata2* gene. **c** Model depicting the change in the chromatin architecture around the *Zbtb16* gene

Another important gene with a high degree in the transcription regulation network (Fig. S15) that encodes the zinc finger protein ZBTB16 (also known as PLZF) was also involved in the differential gene-regulatory interactions. The gene expression and chromatin interaction of this gene were dramatically decreased following the ATRA stimulation (Fig. S17). A previous study using ChIA-PET identified three upstream and two downstream anchors with CTCF binding around the *Zbtb16* gene locus in the K562 cell line (Fig. 6a)^39^. To determine whether the chromatin loops found by ChIA-PET changed upon ATRA induction, we performed a 4C analysis using the five ChIA-PET anchors as baits. The result of one 3′ anchor was a disappearance of the chromatin loop between 5′ and 3′ of *Zbtb16*, accompanied by a significant decrease in ZBTB16 mRNA (Figure S17). This result was consistent with the previous knowledge that the 5′ and 3′ loop maintains a high gene expression level^40^. In both the ATRA-treated and control cells, an ATAC-seq peak was observed in the 5′ of *Zbtb16*. Unexpectedly, only the peak in the ATRA-treated cells enriched the PU.1 motif, suggesting that PU.1 may bind to the 5′ anchor following the ATRA treatment. Furthermore, the transcription regulation of the chromatin conformation does not solely exist in GATA2. Based on these results, we propose two models that explain the chromatin structure, transcription factor binding and gene expression alterations during ATRA-induced differentiation in the *Gata2* and *Zbtb16* regions. In the *Gata2* region, PU.1, RUNX1, and GATA2 bind upstream enhancers, maintain the chromatin loops and facilitate transcription (Fig. 6b). The ATRA treatment causes the loss of transcription factor binding, disrupts chromatin loops and inhibits *Gata2* transcription. In the *Zbtb16* region, the 5′ and 3′ loop maintains the continuous transcription of *Zbtb16* in the control cells. Following the ATRA treatment, in contrast to *Gata2*, new binding of PU.1 breaks the chromatin loop and inhibits transcription (Fig. 6c).

## Discussion

TADs are stable structural and functional units of a genome, and have been shown to maintain their positions in different cell types during ES cell differentiation or following drug treatment^6,41^. We also obtained similar TAD classification results in the control and ATRA-treated cells. However, a small proportion of TADs exhibited significant changes in the internal chromatin interaction frequency (Fig. 2c), indicating that the local chromatin structure within the TADs was altered upon ATRA induction. We observed that the DEGs tend to locate in certain TADs with distinct internal chromatin interaction changes, suggesting the existence of a positive correlation between transcription and chromatin interaction frequency. Our data showed that the internal chromatin interaction frequency in TADs could also be cell type specific and that TADs with distinct changes may be crucial for the differentiation process.

Chromatin interactions maintain special contact between genes and distal regulatory elements, such as enhancers and promoters, ensuring the rapid and precise regulation of gene expression^7,42^. Previously reported myeloid differentiation marker genes, such as *cd38*^43^ and *ncf2*^44^, were found to be involved in differential gene-regulatory interactions, indicating that specific changes in chromatin interactions may regulate the expression of important genes. Nevertheless, although genes with differential gene-regulatory chromatin interactions showed a higher proportion of differential expression, several DEGs did not exhibit differential interactions. For example, the retinoic acid receptor gene *rara*^45^ was upregulated upon the induction, but its promoter did not show any differential interactions with regulatory regions, suggesting that it was be regulated locally (Fig. S13). Interestingly, the ChIA-PET data revealed that widespread promoter-centered chromatin interactions associated with RNA polymerase II facilitate gene expression;^12^ however, PML/RARα-mediated chromatin interactions have been shown to inhibit downstream gene expression, suggesting that the effects of chromatin interactions on transcription are complex. In our data, most gene-regulatory interactions were positively correlated with gene expression (Fig. 3d, e), indicating that transcription-activated chromatin interactions are in the majority. However, we also found 4 downregulated genes in Gain interactions and 13 upregulated genes in Loss interactions, suggesting that a small proportion of transcription-repressed interactions may exist.

One of critical features of leukemia is differentiation blockage^46,47^. ATRA releases this blockage, induces cells into granulocyte differentiation and finally cures the disease^48^. Transcription factors have been shown to be key determinants of differentiation fates. Among the TFs, GATA2 is preferentially transcribed in hematopoietic stem cells (HSCs) and is crucial for the proliferation and self-renewal of HSCs^49,50^. The GATA2 gain-of-function mutant was shown to be associated with the AML transformation into CML^51^. In our study, both the expression level and genomic binding of GATA2 were downregulated upon ATRA induction. Furthermore, in the transcription regulation network, GATA2 exhibited a regulatory potential for nearly all DEGs, suggesting that GATA2 is important for ATRA-induced cell differentiation. The promoter–enhancer loop and 5′–3′ loop were the basic types of chromatin loops that facilitated gene transcription^52^. The loop structure around *Gata2* and *Zbtb16* in the control cells was consistent with these two types, showing the applicability of our differential gene-regulatory interaction analysis.

In summary, we established an experimental and computational framework integrating multiple omics data to study the relationship between the chromatin conformation and transcription regulation (Fig. S18). Applying this framework to the ATRA-induced leukemia differentiation process, we propose that specific chromatin conformation alterations affect pivotal gene transcription and chromatin conformation and provide a novel type of potential drug targets for disease therapy.

## Materials and methods

### Cell culture and ATRA induction

HL60 cells were purchased from China Infrastructure of Cell Line Resources (Peking Union Medical College, Beijing, China). The cells were maintained in RPMI-1640 medium (Gibco, USA) supplemented with 10% fetal bovine serum (FBS, Gibco, USA), 50 units/ml penicillin and streptomycin (Gibco, USA), and Non-Essential Amino Acids (Gibco, USA) as previously described^53^.

For the granulocytic differentiation, 2 × 10^5^/ml HL-60 cells were treated with 1 μM ATRA (1 mM stock in ethanol, Sigma, USA) for 4 days (referred to as the ATRA group) as previously described^54^. Cells treated with an equal amount of ethanol were referred to as the control group. On day 2, the medium was changed, and ATRA/ethanol was added.

For the morphological assessment, the HL-60 cells were harvested, fixed in 4% paraformaldehyde (Thermo Fisher, USA) at room temperature (RT), and stained with 2 μg/ml DAPI (Sigma, USA) in 1× PBS for 15 min at RT. After washing twice with 1× PBS, one droplet of cells was placed on Superfrost slides (Thermo Fisher, USA), which were mounted with 0.17 mm cover slides (Thermo Fisher, USA) by Prolong Gold anti-fade mounting media (Thermo Fisher, USA). The cell nucleus morphology was observed under a Caul Zuess LSM780 confocal scanning laser microscope (CLSM) equipped with ×63 APO PLAN oil lens and 405 nm diode laser. The images were processed using FIJI software. The granulocytic differentiation and cell surface marker were analyzed by flow cytometry (LSRfortessa, Becton–Dickinson, USA) as previously described^43^. In brief, ~1 × 10^6^ cells were washed with MACS buffer (1× PBS with 1 mM EDTA and 1% FBS) and stained with PE-conjugated anti-CD11b and APC-conjugated anti-CD14 antibodies (Becton–Dickinson, USA) for 60 min at 4 °C in the dark. The cells were then washed two times with MACS buffer and analyzed by flow cytometry with a minimum acquisition of 10,000 events. Non-binding mouse PE and APC-labeled IgG (Becton–Dickinson, USA) were used as controls. The flow cytometry data were processed using FlowJo software.

### Real-time reverse transcription-polymerase chain reaction

The gene expression was examined by performing an RT-PCR analysis using previously described protocols^55^. Total RNA was extracted using TRIZOL reagent and reversely transcribed using a first-strand cDNA synthesis kit (Takara, China) according to the manufacturer’s protocol. Real-time RT-PCR was performed using SYBR Green II (Takara, China) and inventoried assay on CFX96 Real-Time PCR platform (Bio-Rad, USA). The housekeeping gene glyceraldehyde 3-phosphate dehydrogenase (GAPDH) was used as an internal control for normalization. The gene-specific primers were synthesized by Sangon Biotech (China), and their sequences are listed in Supplementary File S4.

### Hi-C library construction and data analysis

The in situ Hi-C library construction of the control and ATRA-treated cells was performed as previously described^56^ with some modifications. Briefly, the cells were treated with 1% formaldehyde to crosslink protein–protein and protein–DNA in the cells, and then, the cells were suspended using lysis buffer (50 mM HEPES-KOH, 150 mM NaCl, 1 mM EDTA, 1% Triton X-100, and 0.1% SDS). The genome was then digested by enzyme HaeIII into fragments with blunt-ends. The blunt-ends of the DNA fragments were treated with adenine and ligated with bridge linker containing biotin for 4 h at 16 °C. The unligated DNA fragments were digested with exonuclease (NEB). Next, the cells were digested by Proteinase K (Ambion) overnight, and the DNA was purified using phenol–chloroform (Solarbio) extraction with ethanol precipitation. Then, the DNA was fragmented using an S220 Focused-ultrasonicator (Covaris), and the biotin-labeled DNA fragments were pulled by streptavidin coated Dynabeads M280 (Thermo Fisher). The library from the beads was subjected to Illumina sequencing and amplification by PCR. After purification with the AMPure XP beads (Beckman, Germany), the libraries were sequenced on an Illumina HiSeq 2500 sequencer.

The Hi-C data analysis was performed as follows. First, the reads were trimmed by bridge linker (Sequence: CGCGATATCTTATCTGACT or GTCAGATAAGATATCGCGT), and the 5′ fragments were maintained if the full linker separated the reads into two fragments. Second, the trimmed reads were mapped to the human genome reference hg19, and duplications were removed. Third, the distance threshold of the paired reads was estimated by strand information, and the span of the paired reads and strand information were combined to classify the interaction pairs into full segments (no ligation occurred within the pairs), self-ligations, inter-chromosomal ligations, and intra-chromosomal ligations. The threshold for intra-chromosomal ligations was ~3 kb for a 4-base pair restriction enzyme. The definitions of full segments and self-ligation are as previously described (6), and detailed statistical information is provided in Supplementary File S3.

For the Hi-C data correction, we used the iterative correction method (ICE)^57^ to correct systematic biases. The compartment pattern analysis was performed as previously described^58^. In brief, interaction matrices at a 40 kb resolution were first generated, and a principal component analysis was then performed. The first principle component was used to define the compartment pattern. Replicate data were used to classify the compartments separately, and compartment changes were defined using both replicates. For the TAD classification, we used Clustering-based Hi-C Domain Finder (CHDF) as previously described^29^.

### Calculation of fold changes in TAD interactions

To calculate the fold changes in the TAD internal and external Hi-C count upon ATRA induction, we first calculated the fold changes between the replicates. For each TAD, the fold changes in the replicates of the control and ATRA-treated cells were combined to generate a background distribution (internal and external fold changes were calculated separately). Then, the fold changes between the ATRA-treated and control cells were introduced into the background distribution to obtain a *p*-value based on their position in the background distribution. TADs with a *p*-value < 0.05 in both replicates were defined as significantly changed TADs. The results of all TADs are shown in Supplementary File S5.

### Differential gene-regulatory interaction analysis

The chromosomal sequencing was divided into 40 kb bins. Once the H3K4me3 peaks attained by ChIP-seq were located within the spread of the TSS in an expressed gene, the bin in which the H3K4me3 peak was located was labeled the gene region. In addition, the H3K27ac peaks that were not located within the spread of the TSS as regulatory sequences were assigned into bins labeled regulatory regions.

We generated a chromatin interaction matrix at a 40 kb resolution using the Hi-C data, and the counts in the corresponding element of the matrix indicate the interaction intensity between the gene and regulatory regions. For example, if the *i*th bin is a gene region and the *j*th bin is a regulatory region, counts of [*i,j*] represent the gene-regulatory interaction. Gene-regulatory chromatin interactions within the same TADs were considered input in the differential interaction analysis (Supplementary File S5). A differential interaction analysis was performed using the MA-plot-based method with a random sampling model. In brief, we assumed that the counts of specific interactions follow a binomial distribution. Let C1 and C2 denote the counts of a specific gene-regulatory interaction obtained from control and ATRA-treated cells with Ci~binomial (*ni*, *pi*), *i* = 1, 2, where *ni* denotes the total number of Hi-C counts, and *pi* denotes the probability of a count originating from that gene-regulatory interaction. We define *M* = (log2C1−log2C2)/2, and *A* = (log2C1 + log2C2)/2. Under the random sampling assumption, the conditional distribution of *M* given that *A* = *a* (*a* is an observation of *A*) follows an approximate normal distribution. For each gene-regulatory interaction on the MA-plot, we perform hypothesis testing of H0: p1 = p2 vs. H1: p1 ≠ p2. Then, a *p*-value is assigned based on the conditional normal distribution. The analysis was performed using the DEGseq package in R 3.3.1 with the parameter setting ‘MARS’^59^. The data replicates were combined to improve the accuracy. The differential gene-regulatory interactions associated with the DEGs are shown in Supplementary File S6.

### RNA-Seq library construction and data analysis

Total RNA was extracted from the control and ATRA-treated cells using TRIZOL (Ambion, USA) as previously described^60^. The library construction and sequencing were performed by ANOROAD (China).

For the RNA-Seq analysis, the adapter sequences were first removed. Ribosomal RNA reads, if any, were filtered using Bowtie. After the quality filtering and adapter removal steps, the reads were aligned to a transcriptome and quantified using RSEM v.1.2.7. The annotation file was downloaded from the University of California, Santa Cruz (UCSC) genome browser, human hg19 assembly. The differential gene expression was calculated using the Deseq2 version 1.4.5 package according to the mean value of the gene-wise dispersion estimates^61^. The differential expression analysis results are shown in Supplementary File S6. To identify the significantly DEGs, we used an adjusted *p*-value of 0.01 and a log2-fold change>0.9. A Gene Ontology analysis was performed using DAVID. The RNA-seq plots were confirmed using the ggplot2 package in R 3.3.1.

### ChIP-seq library construction and data analysis

The ChIP-seq analysis of the control and ATRA-treated HL-60 cells was performed as previously described^40,41^. The cells were treated with 1% formaldehyde to crosslink. Then, the cell membrane was broken using lysis buffer (50 mM HEPES-KOH, 150 mM NaCl, 1 mM EDTA, 1% Triton X-100, 0.1% sodium deoxycholate, and 1% SDS). Chromatin were resuspended using FA lysis buffer (50 mM HEPES-KOH, 150 mM NaCl, 1 mM EDTA, 1% Triton X-100, 0.1% sodium deoxycholate, and 0.1% SDS) and fragmented by an ultra-sonication processor (Cole-Parmer, USA). Immunoprecipitation was performed overnight using Dynabeads (Thermo Fisher) pre-incubated with anti-H3K4me3 and H3K27ac antibodies (Abcam, England). After washing and purifying the DNA, we constructed libraries using a TruePrep DNA Library Prep Kit (Vazyme, China) according to the manufacturer’s protocol. Fragmented genomic DNA was also used to construct the libraries as input for ChIP-seq. The libraries were sequenced using an Illumina HiSeq 2500 sequencer.

For the ChIP-Seq analysis, the adapter sequences were first removed. Then, the reads were aligned to the human hg19 assembly using Bowtie. The histone modification peaks were called by MACS2^62^ using the parameter setting ‘-g hs --nomodel --broad’. All peaks are listed in GEO data set mentioned below. The peaks found in both replicates (using bedtools with a 1 bp minimal overlap) were kept as convincing peaks. Then, the convincing peaks in the control and ATRA-treated cells were compared to distinguish the ATRA-specific, control-specific and overlapping peaks. The peak comparisons were performed using intersectBed software in bedtools.

### ATAC-seq library construction and data analysis

The ATAC-seq libraries of the control and ATRA-treated cells were prepared as previously described^63^. Briefly, the samples were lysed in lysis buffer (10 mM Tris-HCl (pH 7.4), 10 mM NaCl, 3 mM MgCl_2_ and NP-40) for 10 min on ice to prepare the nuclei. Immediately after lysis, the nuclei were spun to remove the supernatant. The nuclei were then incubated with the Tn5 transposome and tagmentation buffer at 37 °C for 30 min (Vazyme, China). After the tagmentation, the stop buffer was directly added to the reaction to end the tagmentation. PCR was performed to amplify the library in 12 cycles. After the PCR reaction, the libraries were purified with 1.2× AMPure beads (Beckman, Germany). The libraries were sequenced using an Illumina HiSeq 2500 sequencer.

The removal of the adapter sequences and mapping were performed as described for the ChIP-seq library construction and data analysis. The ATAC-seq peaks were called by MACS2 using the default parameters. All peaks are listed in GEO data set mentioned below. The peak comparisons were performed as described for the ChIP-seq library construction and data analysis. To identify the sequence motif enriched in the ATAC-seq peaks, findMotifsGenome.pl in the HOMER program^64^ was used. AnnotatePeaks.pl was used to identify specific peaks that contain certain motifs. A GREAT analysis of the ATAC-seq peaks was performed as previously described^65^. The GREAT results are shown in Supplementary File S7.

### 4C-seq library construction and data analysis

The 4C-seq libraries of the control and ATRA-treated cells were prepared as previously described^66^. First, the cells were treated with 1% formaldehyde to crosslink, and the nuclei were isolated via resuspension in lysis buffer (500 μl 10 mM Tris-HCl pH 8.0, 10 mM NaCl, 0.2% Igepal CA-630 and 50 μl protease inhibitors). Then, the nuclei were washed with 1× NEBuffer2 (NEB, England) and treated with 0.3% SDS at 65 °C. HindIII digestion was performed at 37 °C overnight, and then, a proximity ligation was performed at 4 °C for 4 h. After reversing the cross-linking with a Proteinase K (Ambion, USA) treatment, the DNA was purified using a phenol–chloroform (Solarbio) extraction with ethanol precipitation. The second digestion step was performed using DpnII overnight. Then, we performed a second ligation and ethanol precipitation to extract the DNA. The DNA was finally purified using a QIAquick PCR purification kit (QIAGEN, Germany) according to the manufacturer’s protocol. After the PCR reaction, we purified the 4C library using AMPure beads (Beckman, Germany). The libraries were sequenced using an Illumina HiSeq 2500 sequencer. The primer sequences for GATA2 and ZBTB16 are listed in Supplementary File S4.

For the 4C-seq data analysis, the adapter sequences were first removed using cutadapt software in SAMtools. Bowtie was used to map the reads to the human hg19 assembly. Then, the mapped data were processed using the r3Cseq package in R 3.3.1 using RPM normalization.

### Fluorescent in situ hybridization

The GATA2 interaction genomic loci were viewed in the UCSC genome browser, and the BAC ends track was enabled to facilitate the determination of the suitable BAC clone. CTD-2306G6 (containing the GATA2 promoter, chr3:128212030–128214030) and RP11-722C17 (containing the GATA2 enhancer, chr3: 128309790–128334446) were purchased from Life Technologies. The BAC clones were isolated using an Epicenter BACMAX kit (Epicenter, USA) and then labeled with Alexafluor488 (GATA2 enhancer) and Alexafluor594 (GATA2 promoter) by nick translation. Briefly, 1 μg BAC clone was mixed with 2U *E. coli* DNA Pol I (NEB, England), 100 mU DNase I (NEB, England), 2.5 mM dNTP mix, and 1 mM fluorescent labeled dUTP and incubated at 15 °C for 2 h. The reaction was terminated by adding 2 μl 0.5 M EDTA, followed by an incubation at 85 °C for 20 min. Then, the probes were isolated by ethanol precipitation and resuspended in hybridization buffer (50% formamide, 2× SSC, and 10% Dextran Sulfate). For the hybridization steps, briefly, the HL-60 cells were fixed in 4% paraformaldehyde for 15 min at room temperature, permeated for 30 min in 0.1% TritonX-100, and soaked in 20% Glycerin for 30 min. The cells were freeze/thawed three times in liquid nitrogen and then treated with 0.1 M HCl. After washing with PBS, the cells were incubated with 100 μg/ml RNase A at 37 °C for 1 h to remove the RNA. Finally, the cells were kept in 50% formamide and 2× SSC and prepared for FISH. In total, 20 ng of each prepared probe were mixed with 10 μl hybridization buffer. The cells were denatured at 83 °C for 5 min and hybridized at 37 °C for 12 h. After the hybridization, the cells were washed with 0.4% CA-630 in 2× SSC three times for 5 min each. Finally, the cells were mounted with Mowiol mounting media and 2 μg/ml DAPI and imaged under an LSM 780 confocal microscope.

All images were processed by FIJI, and the Pearson Correlation Coefficients were calculated by the ImageJ plugin JACOP.

### Sequencing data

The RNA-seq/Hi-C/ChIP-seq/ATAC-seq data discussed in this manuscript have been deposited in NCBI’s Gene Expression Omnibus and are accessible through GEO Series accession number GSE93997. The secure token evknkasaxlgllof must be used to access the data set.

## Electronic supplementary material

Instructions of supplementary files

Supplementary figures

Hi-C library statistics

Primer sequence

Interaction foldchanges of TADs

Differential gene-regulatory analysis of expressed genes

GREAT analysis of specific ATAC-seq peaks

## References

[1] Cremer C, Cremer T, Gray JW (1982). Induction of chromosome damage by ultraviolet light and caffeine: correlation of cytogenetic evaluation and flow karyotype. Cytometry.

[2] Bolzer A (2005). Three-dimensional maps of all chromosomes in human male fibroblast nuclei and prometaphase rosettes. PLoS Biol..

[3] Shopland LS (2006). Folding and organization of a contiguous chromosome region according to the gene distribution pattern in primary genomic sequence. J. Cell Biol..

[4] Peric-Hupkes D (2010). Molecular maps of the reorganization of genome-nuclear lamina interactions during differentiation. Mol. Cell.

[5] Chaumeil J, Le Baccon P, Wutz A, Heard E (2006). A novel role for Xist RNA in the formation of a repressive nuclear compartment into which genes are recruited when silenced. Genes Dev..

[6] Jin F (2013). A high-resolution map of the three-dimensional chromatin interactome in human cells. Nature.

[7] Rao SSP (2014). A 3D map of the human genome at kilobase resolution reveals principles of chromatin looping. Cell.

[8] Heger P, Marin B, Bartkuhn M, Schierenberg E, Wiehe T (2012). The chromatin insulator CTCF and the emergence of metazoan diversity. Proc. Natl Acad. Sci. USA.

[9] Dixon JR (2012). Topological domains in mammalian genomes identified by analysis of chromatin interactions. Nature.

[10] Le Dily F (2014). Distinct structural transitions of chromatin topological domains correlate with coordinated hormone-induced gene regulation. Genes Dev..

[11] Fullwood MJ (2009). An oestrogen-receptor-alpha-bound human chromatin interactome. Nature.

[12] Li G (2012). Extensive promoter-centered chromatin interactions provide a topological basis for transcription regulation. Cell.

[13] Hsu PY (2013). Amplification of distant estrogen response elements deregulates target genes associated with tamoxifen resistance in breast cancer. Cancer Cell.

[14] Nora EP (2012). Spatial partitioning of the regulatory landscape of the X-inactivation centre. Nature.

[15] Gibcus JH, Dekker J (2013). The hierarchy of the 3D genome. Mol. Cell.

[16] Nora EP, Dekker J, Heard E (2013). Segmental folding of chromosomes: a basis for structural and regulatory chromosomal neighborhoods?. Bioessays.

[17] Javierre BM (2016). Lineage-specific genome architecture links enhancers and non-coding disease variants to target gene promoters. Cell.

[18] Tohno Y, Tohno S, Tanaka Y (1995). Chromatin loop size in human leukemia (HL-60) cells. J. Electron Microsc. (Tokyo).

[19] Kim A, Dean A (2012). Chromatin loop formation in the β-globin locus and its role in globin gene transcription. Mol. Cells.

[20] Fraser J (2009). Chromatin conformation signatures of cellular differentiation. Genome Biol..

[21] Rousseau M (2014). Classifying leukemia types with chromatin conformation data. Genome Biol..

[22] Zhang J (2016). Distal regulation of c-myb expression during IL-6-induced differentiation in murine myeloid progenitor M1 cells. Cell Death Dis..

[23] Mazumdar C, Majeti R (2017). The role of mutations in the cohesin complex in acute myeloid leukemia. Int. J. Hematol..

[24] Dowen JM (2014). Control of cell identity genes occurs in insulated neighborhoods in mammalian chromosomes. Cell.

[25] Griffin J, Munroe D, Major P, Kufe D (1982). Induction of differentiation of human myeloid leukemia cells by inhibitors of DNA synthesis. Exp. Hematol..

[26] Barber N, Belov L, Christopherson RI (2008). All-trans retinoic acid induces different immunophenotypic changes on human HL60 and NB4 myeloid leukaemias. Leuk. Res..

[27] Olins AL, Olins DE (2004). Cytoskeletal influences on nuclear shape in granulocytic HL-60 cells. BMC Cell Biol..

[28] Sexton T (2012). Three-dimensional folding and functional organization principles of the Drosophila genome. Cell.

[29] Wang Y, Li Y, Gao J, Zhang MQ (2015). A novel method to identify topological domains using Hi-C data. Quant. Biol..

[30] Barutcu AR (2015). Chromatin interaction analysis reveals changes in small chromosome and telomere clustering between epithelial and breast cancer cells. Genome Biol..

[31] Barutcu AR (2016). RUNX1 contributes to higher-order chromatin organization and gene regulation in breast cancer cells. Biochim. Biophys. Acta.

[32] Heidari N (2014). Genome-wide map of regulatory interactions in the human genome. Genome Res..

[33] Sanyal A, Lajoie BR, Jain G, Dekker J (2012). The long-range interaction landscape of gene promoters. Nature.

[34] West AG, Fraser P (2005). Remote control of gene transcription. Hum. Mol. Genet..

[35] Zhang Y (2013). Chromatin connectivity maps reveal dynamic promoter-enhancer long-range associations. Nature.

[36] Rosenbauer F, Tenen DG (2007). Transcription factors in myeloid development: balancing differentiation with transformation. Nat. Rev. Immunol..

[37] Bovolenta LA, Acencio ML, Lemke N (2012). HTRIdb: an open-access database for experimentally verified human transcriptional regulation interactions. BMC Genom..

[38] Yamazaki H (2014). A remote GATA2 hematopoietic enhancer drives leukemogenesis in inv(3)(q21; q26) by activating EVI1 expression. Cancer Cell.

[39] Tang Z (2015). CTCF-mediated human 3D genome architecture reveals chromatin topology for transcription. Cell.

[40] Tan-Wong SM (2012). Gene loops enhance transcriptional directionality. Science.

[41] Dixon JR (2015). Chromatin architecture reorganization during stem cell differentiation. Nature.

[42] Tark-Dame M (2014). Depletion of the chromatin looping proteins CTCF and cohesin causes chromatin compaction: insight into chromatin folding by polymer modelling. PLoS Comput. Biol..

[43] Tee MK, Vigne JL, Taylor RN (2006). All-trans retinoic acid inhibits vascular endothelial growth factor expression in a cell model of neutrophil activation. Endocrinology.

[44] Vrba J, Trtkova K, Ulrichova J (2011). HDAC inhibitors sodium butyrate and sodium valproate do not affect human ncor1 and ncor2 gene expression in HL-60 cells. Biomed. Pap. Med. Fac. Univ. Palacky Olomouc Czech Repub..

[45] Yoshida H (1996). Accelerated degradation of PML-retinoic acid receptor alpha (PML-RARA) oncoprotein by all-trans-retinoic acid in acute promyelocytic leukemia: possible role of the proteasome pathway. Cancer Res..

[46] Jiang G, Albihn A, Tang T, Tian Z, Henriksson M (2008). Role of Myc in differentiation and apoptosis in HL60 cells after exposure to arsenic trioxide or all-trans retinoic acid. Leuk. Res..

[47] Martino V (2009). Down-regulation of HOXA4, HOXA7, HOXA10, HOXA11 and MEIS1 during monocyte-macrophage differentiation in THP-1 cells. Mol. Med. Rep..

[48] Zhang JW, Wang JY, Chen SJ, Chen Z (2000). Mechanisms of all-trans retinoic acid-induced differentiation of acute promyelocytic leukemia cells. J. Biosci..

[49] Tsai FY, Orkin SH (1997). Transcription factor GATA-2 is required for proliferation/survival of early hematopoietic cells and mast cell formation, but not for erythroid and myeloid terminal differentiation. Blood.

[50] Rodrigues NP, Tipping AJ, Wang Z, Enver T (2012). GATA-2 mediated regulation of normal hematopoietic stem/progenitor cell function, myelodysplasia and myeloid leukemia. Int. J. Biochem. Cell Biol..

[51] Zhang SJ (2008). Gain-of-function mutation of GATA-2 in acute myeloid transformation of chronic myeloid leukemia. Proc. Natl Acad. Sci. USA.

[52] Cavalli G, Misteli T (2013). Functional implications of genome topology. Nat. Struct. Mol. Biol..

[53] Ozeki M, Shively JE (2008). Differential cell fates induced by all-trans retinoic acid-treated HL-60 human leukemia cells. J. Leukoc. Biol..

[54] Huang S, Eichler G, Bar-Yam Y, Ingber DE (2005). Cell fates as high-dimensional attractor states of a complex gene regulatory network. Phys. Rev. Lett..

[55] Song G (2013). c-myc but not Hif-1α-dependent downregulation of VEGF influences the proliferation and differentiation of HL-60 cells induced by ATRA. Oncol. Rep..

[56] Van Berkum, N. L. et al. Hi-C: A method to study the three-dimensional architecture of genomes. *J. Vis. Exp.* (2010). https://doi.org./10.3791/1869. 10.3791/1869 PMC3149993 20461051

[57] Imakaev M (2012). Iterative correction of Hi-C data reveals hallmarks of chromosome organization. Nat. Methods.

[58] Lieberman-Aiden E (2009). Comprehensive mapping of long-range interactions reveals folding principles of the human genome. Science.

[59] Wang L, Feng Z, Wang X, Wang X, Zhang X (2010). DEGseq: an R package for identifying differentially expressed genes from RNA-seq data. Bioinformatics.

[60] Wang D (2011). Reprogramming transcription by distinct classes of enhancers functionally defined by eRNA. Nature.

[61] Love MI, Huber W, Anders S (2014). Moderated estimation of fold change and dispersion for RNA-seq data with DESeq2. Genome Biol..

[62] Feng J, Liu T, Zhang Y (2011). Using MACS to identify peaks from ChIP-Seq data. Curr. Protoc. Bioinformatics.

[63] Wu J (2016). The landscape of accessible chromatin in mammalian preimplantation embryos. Nature.

[64] Heinz S (2010). Simple combinations of lineage-determining transcription factors prime cis-regulatory elements required for macrophage and B cell identities. Mol. Cell.

[65] McLean CY (2010). GREAT improves functional interpretation of cis-regulatory regions. Nat. Biotechnol..

[66] Splinter E, de Wit E, van de Werken HJG, Klous P, de Laat W (2012). Determining long-range chromatin interactions for selected genomic sites using 4C-seq technology: from fixation to computation. Methods.

