# Supplementary material for: Alterations of specific chromatin conformation affect ATRA-induced leukemia cell differentiation
Source: Cell Death Dis. 2018 Feb 8;9(2):200. doi: 10.1038/s41419-017-0173-6 (PMC5833835; doi:10.1038/s41419-017-0173-6)
Supplement: Supplementary file 1 — Instructions of supplementary files [file 41419_2017_173_MOESM1_ESM.docx]

**Supplementary Files**

**Supplementary File S1. Instructions of supplementary files**

This file contains instructions of all supplementary files.

**Supplementary File S2. Supplementary figures**

This file contains all supplementary figures and their legends.

**Supplementary File S3. Hi-C library statistics**

This file lists the number of each chromatin interaction type (including Total interaction, Full-segment, Inter-ligation and Intra-ligation) from the control and ATRA-treated Hi-C libraries according to the interaction calling method mentioned in Materials and Methods.

**Supplementary File S4. Primer sequence**

This file lists the primer sequences used in the RT-PCR and 4C experiments. To divide the 4C libraries of the control and ATRA-treated cells, we used the same primer for the HindIII ends and different primers for the DpnII ends.

**Supplementary File S5. Interaction fold changes in TADs**

The first sub-list in this file contains the background distribution generated using the differences between replicates (for detail, see Materials and Methods). The second sub-list in this file lists the internal and external fold change statistics of each TADs. Each row represents a TAD, and the columns represent the start, end, length and interaction fold change between the control and ATRA-treated cells (internal and external fold changes were calculated separately) of TADs. TADs with significant internal/external interaction fold changes are listed in the final two sub-lists.

**Supplementary File S6. Differential gene-regulatory analysis of expressed genes**

The first sub-list in this file lists the DEseq2 results of the RNA-seq data. Each row represents a gene, and the columns represent the mean base, fold change, p-value and adjusted p-value of the genes. The second sub-list only contains the differentially expressed genes. Each row also represents a gene, and the columns represent the fold change and p-value of the differential gene-regulatory interaction analysis and differential expression analysis (For detail, see Materials and Methods).

**Supplementary File S7. GREAT analysis of specific ATAC-seq peaks**

This file lists the GREAT analysis results of the control- and ATRA-specific ATAC-seq peaks. Each row represents a gene expression term, and the columns represent the ranks, p-value and other statistical results of the terms.
