# Supplementary material for: Alterations of specific chromatin conformation affect ATRA-induced leukemia cell differentiation
Source: Cell Death Dis. 2018 Feb 8;9(2):200. doi: 10.1038/s41419-017-0173-6 (PMC5833835; doi:10.1038/s41419-017-0173-6)
Supplement: Supplementary file 2 — Supplementary figures [file 41419_2017_173_MOESM2_ESM.docx]

**Supplementary Figures**


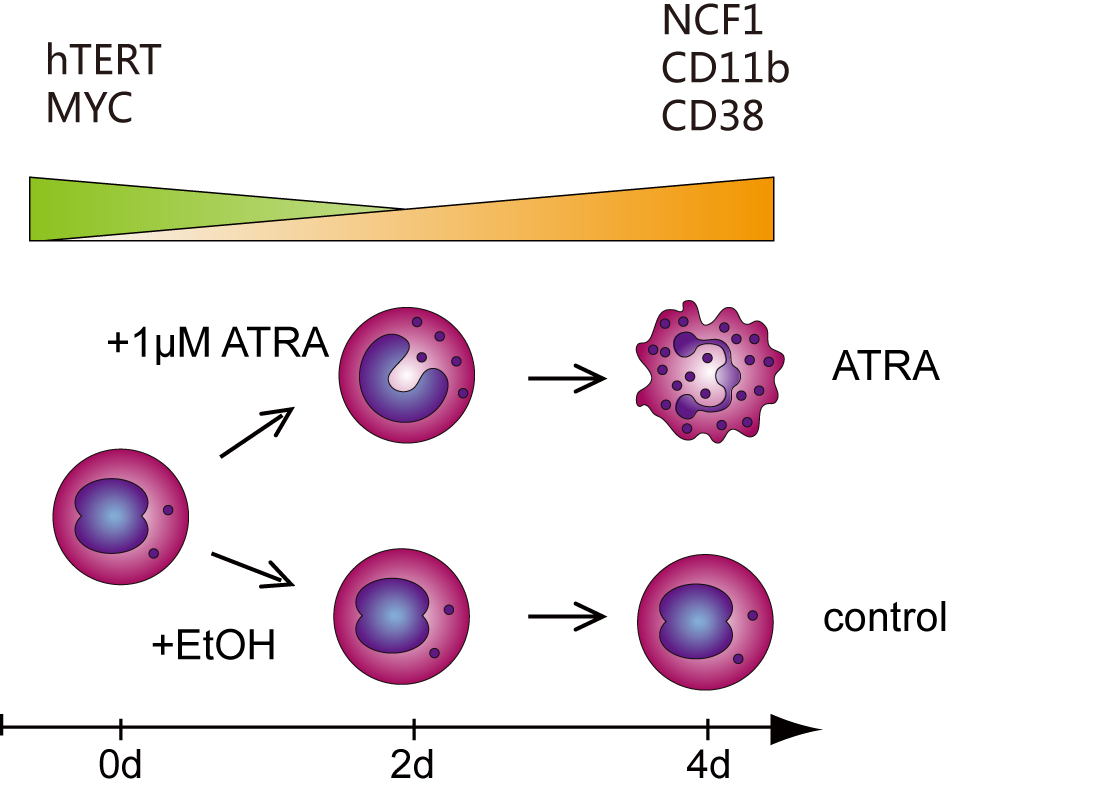


Supplementary Figure S1: Schematic representation of the ATRA-induced HL-60 differentiation process. Cells treated with an equal amount of solvent (ethanol) were used as the control group. Images were obtained from previous reviews.(1)


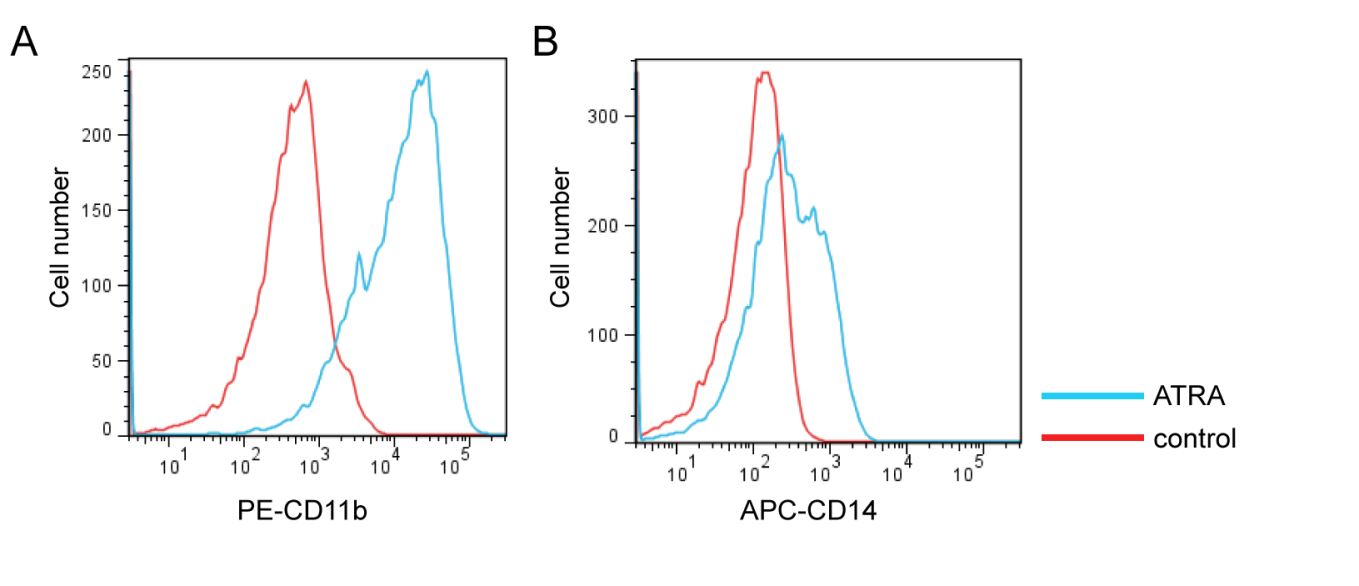


Supplementary Figure S2: Granulocytic differentiation analysis using a cell surface marker. Density distribution of CD11b (A) and CD14 (B) in ATRA-treated cells (blue) and control cells (red) is shown.


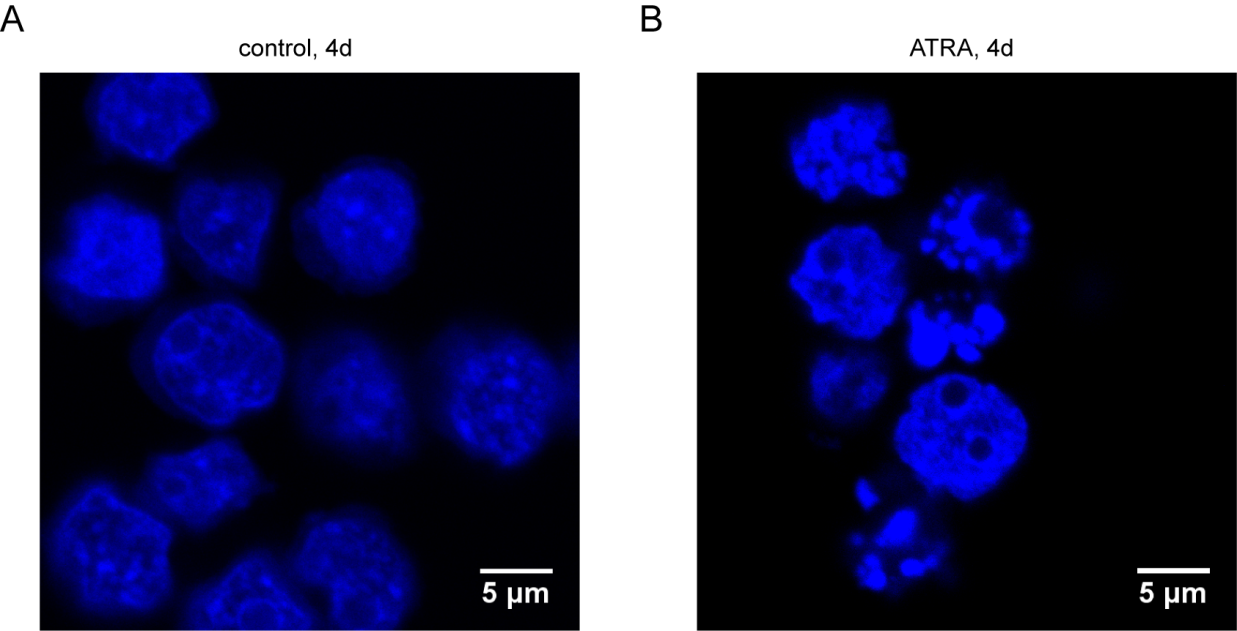


Supplementary Figure S3: Morphological assessments of ATRA-treated (B) and control (A) cells under a microscope. Cell nuclei were stained with DAPI (blue).


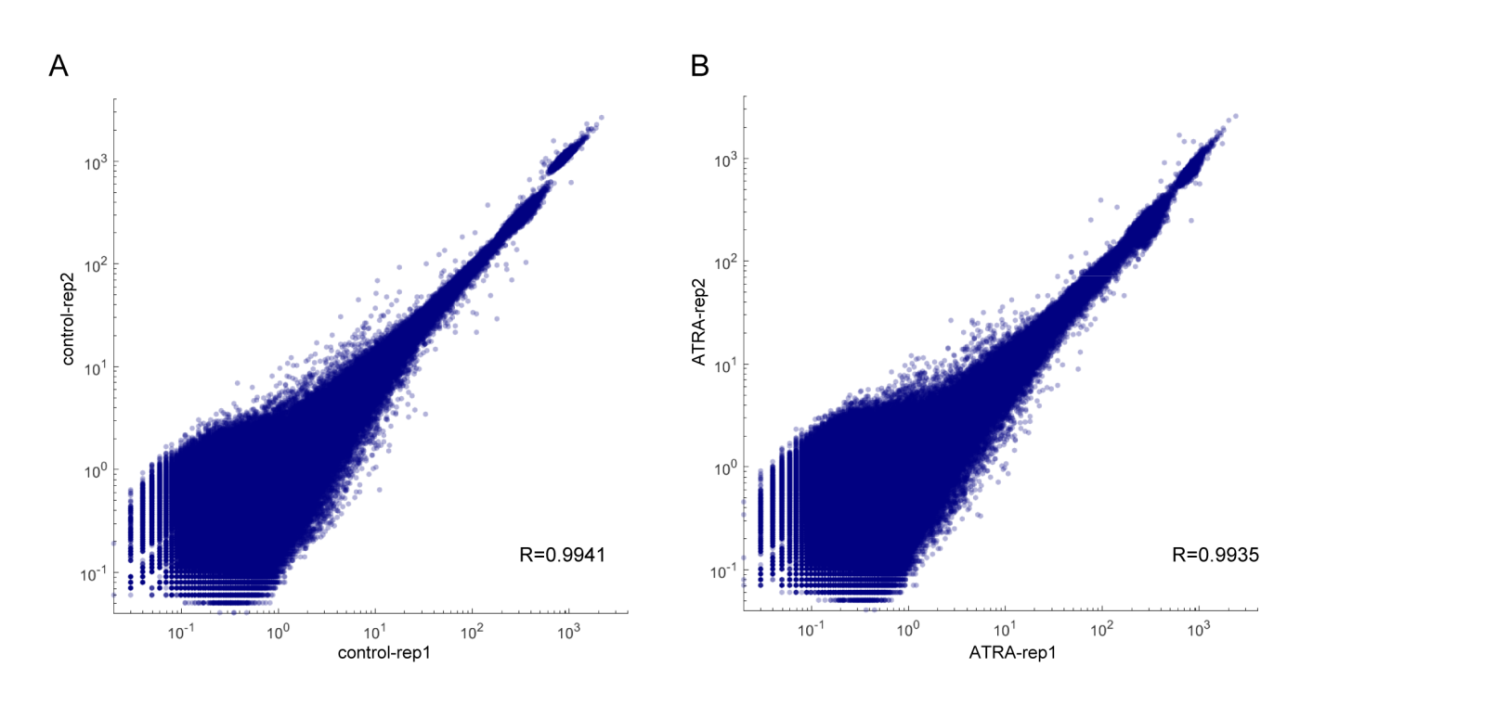


Supplementary Figure S4: Pearson correlation coefficients of genome-wide interactions in biological replicates of control (A) or ATRA-treated (B) cells. 1 Mb sized bins were used.


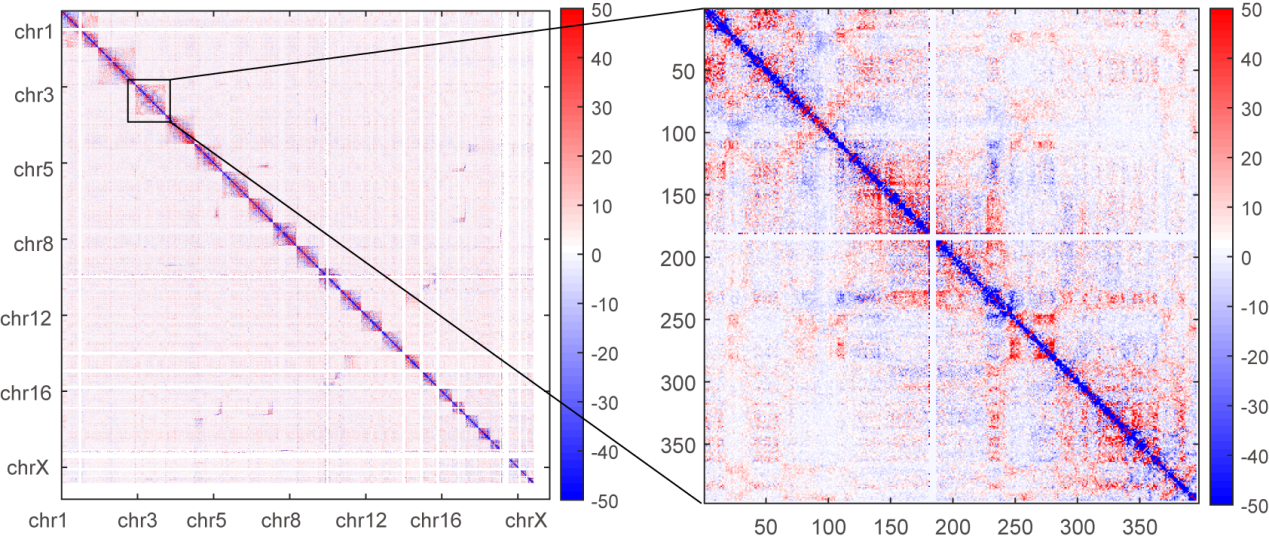


Supplementary Figure S5: (left) Whole-genome differential interactions between ATRA-treated and control HL-60 cells. The red color indicates stronger interactions in ATRA-treated cells, and the blue color indicates stronger interactions in the control cells. Regions with no significant change are indicated in white. (right) Similar to the left, differential interactions in chromosome 3 are shown.


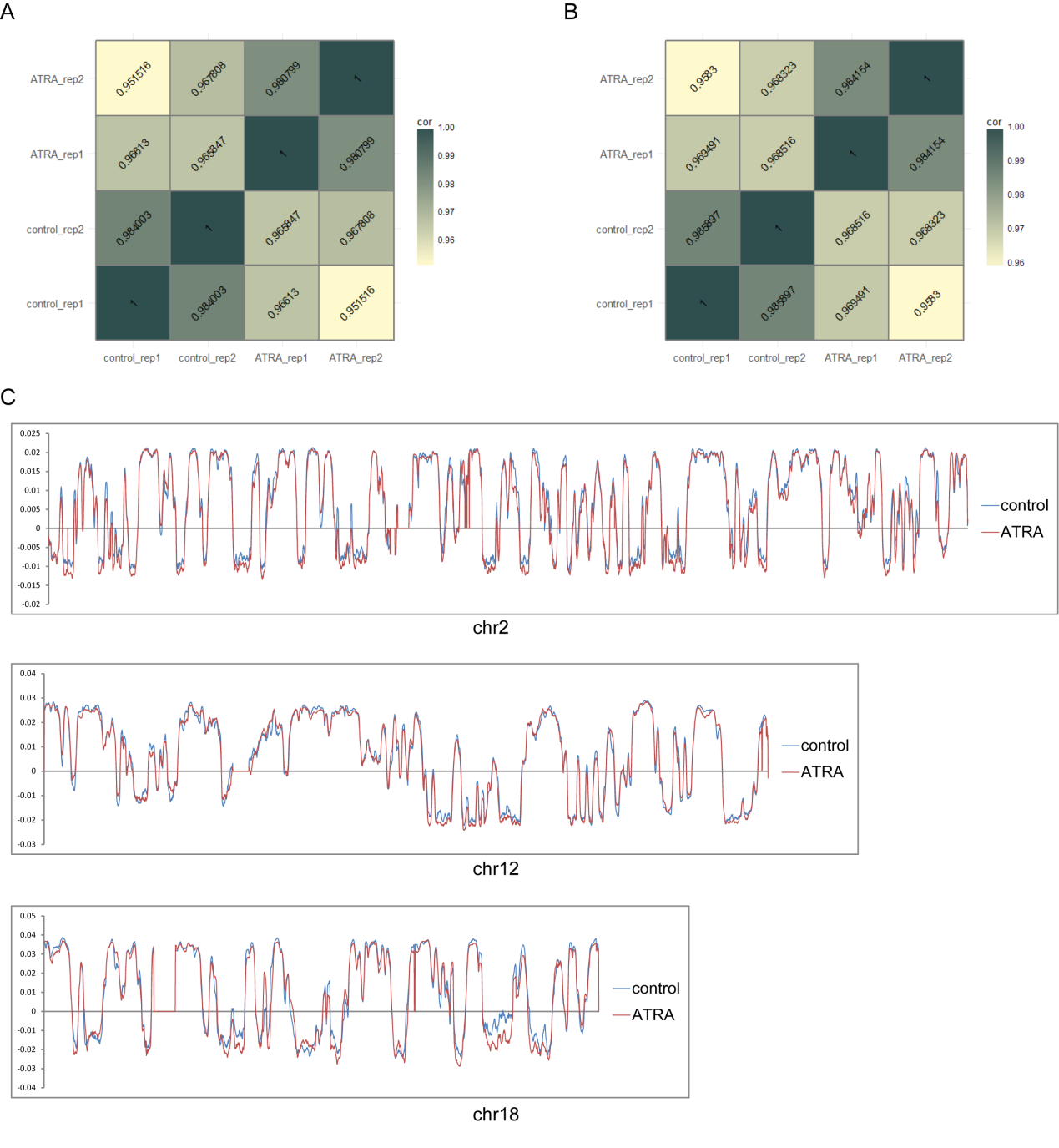


Supplementary Figure S6: Compartment analysis during ATRA-induced differentiation. Pearson (A) and Spearman (B) correlation heatmaps of Eigen values between each sample. (C) Compartment patterns in whole chr2, chr12 and chr18 in the control (blue) and ATRA-treated (red) cells are shown.


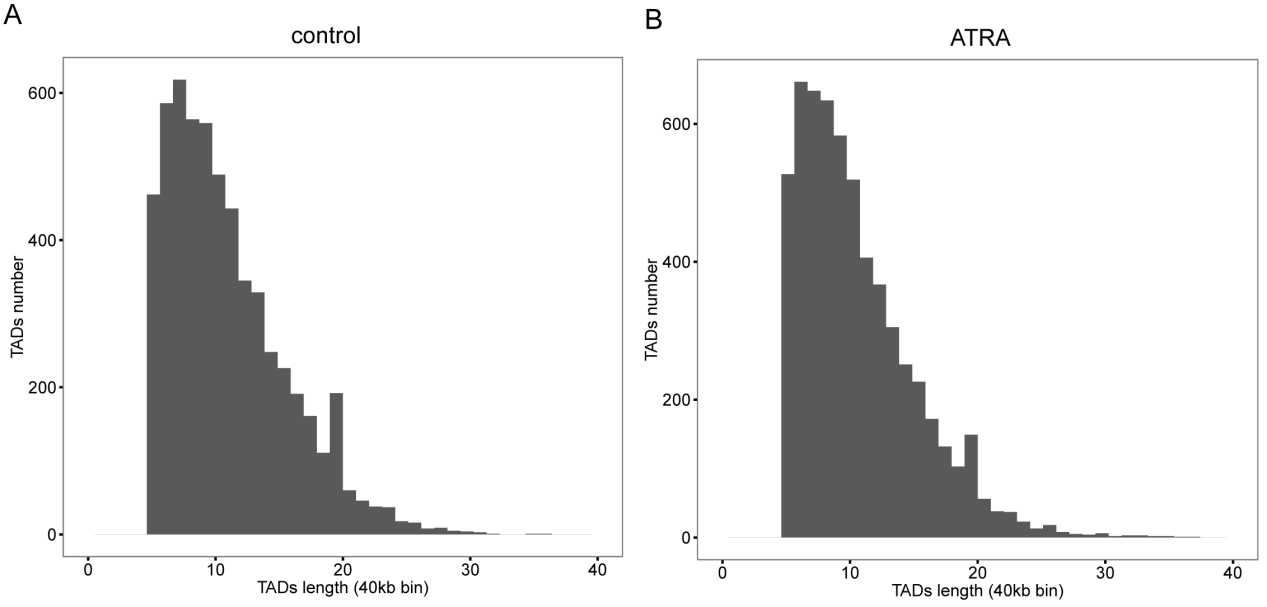


Supplementary Figure S7: Histogram of TAD length in the ATRA-treated (B) and control (A) cells.


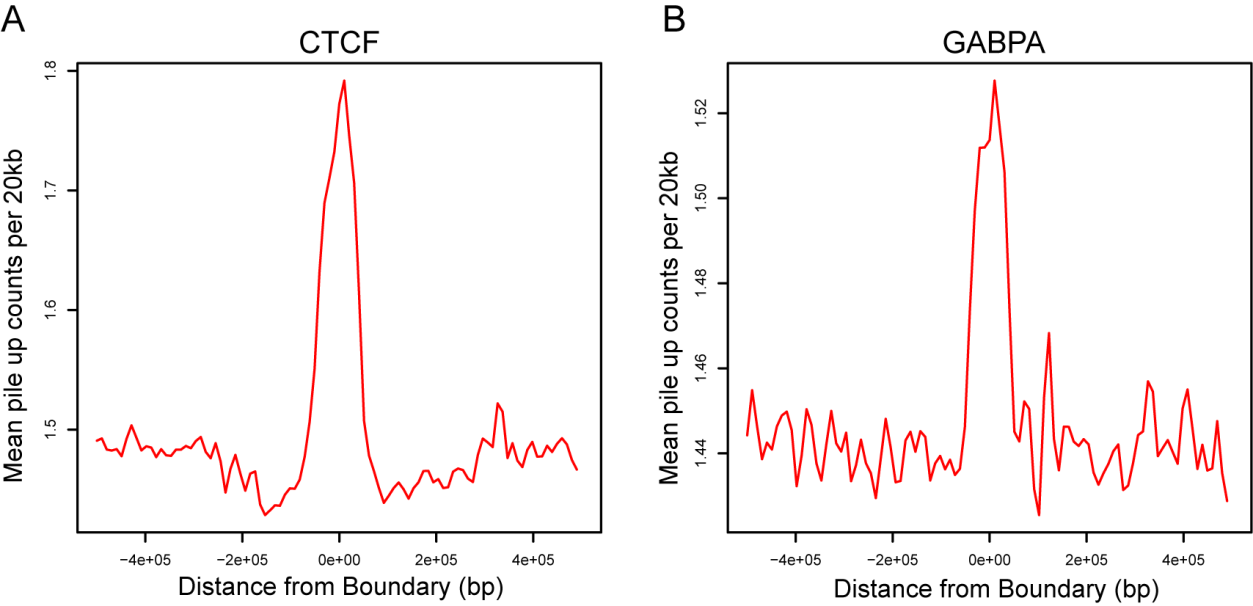


Supplementary Figure S8: Mean pile up count plots of factors enriched at HL-60 TAD boundaries using a 20-kb sliding window for ±500 kb of every TAD boundary. CTCF (A) and GABPA (B) were significantly enriched on TADs boundaries


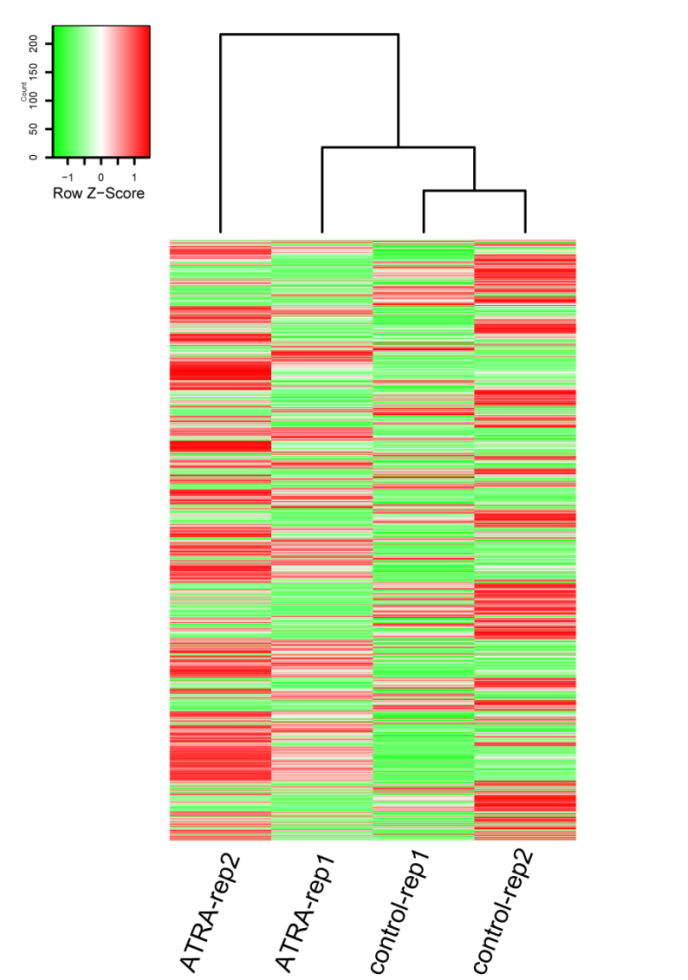


Supplementary Figure S9: Bi-clustering heatmap of all expressed genes. Samples were linked by the dendrogram above to show the similarity in their gene expression patterns.


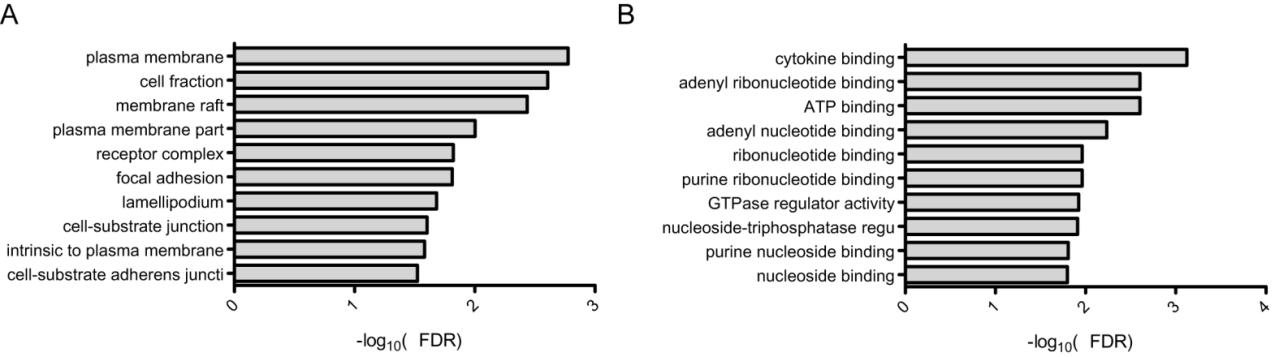
Supplementary Figure S10: GO enrichment results of differentially expressed genes in Cellular Compartment (A) and Molecular Function (B). Top 10 terms ranked by –log10 FDR are shown. FDR: false discovery rate.


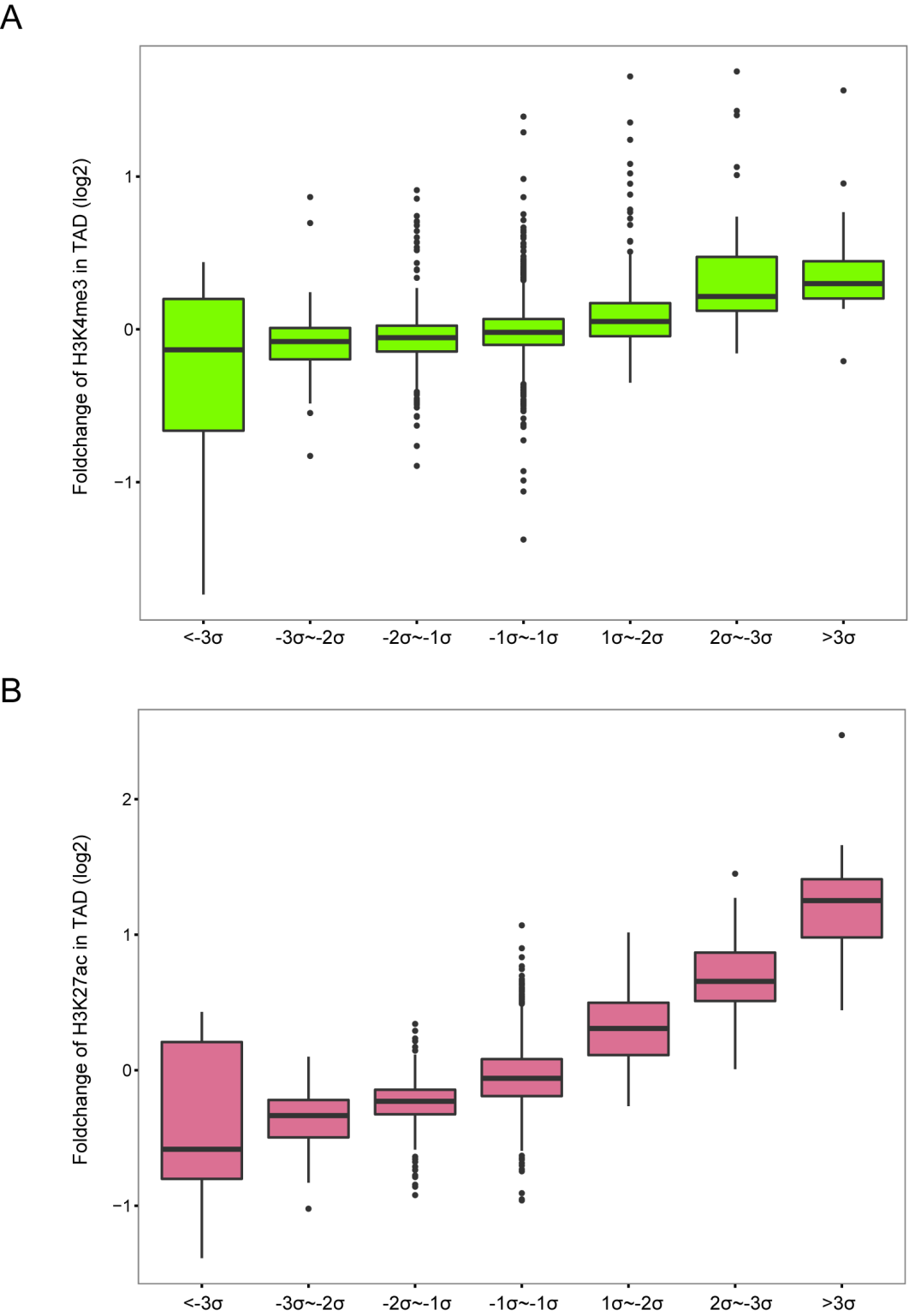


Supplementary Figure S11: Boxplot of log2 H3K4me3 (A) and H3K27ac (B) fold change in TADs. TADs are divided into 7 categories based on their internal Hi-C count fold change as shown in Figure 2C


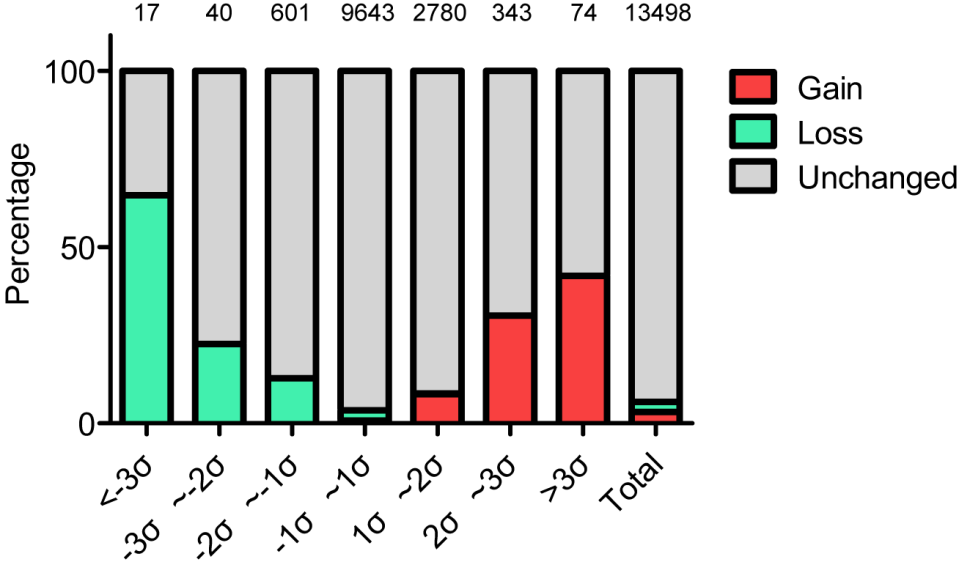


Supplementary Figure S12: Accumulation bar-plot showing the enrichment of differential gene-regulatory interactions in TADs. TADs are divided into 7 categories as shown in Figure 2C, and Total represents all putative gene-regulatory interactions located in TADs. Number of putative gene-regulatory interactions located in each type of TADs is shown above the bar.


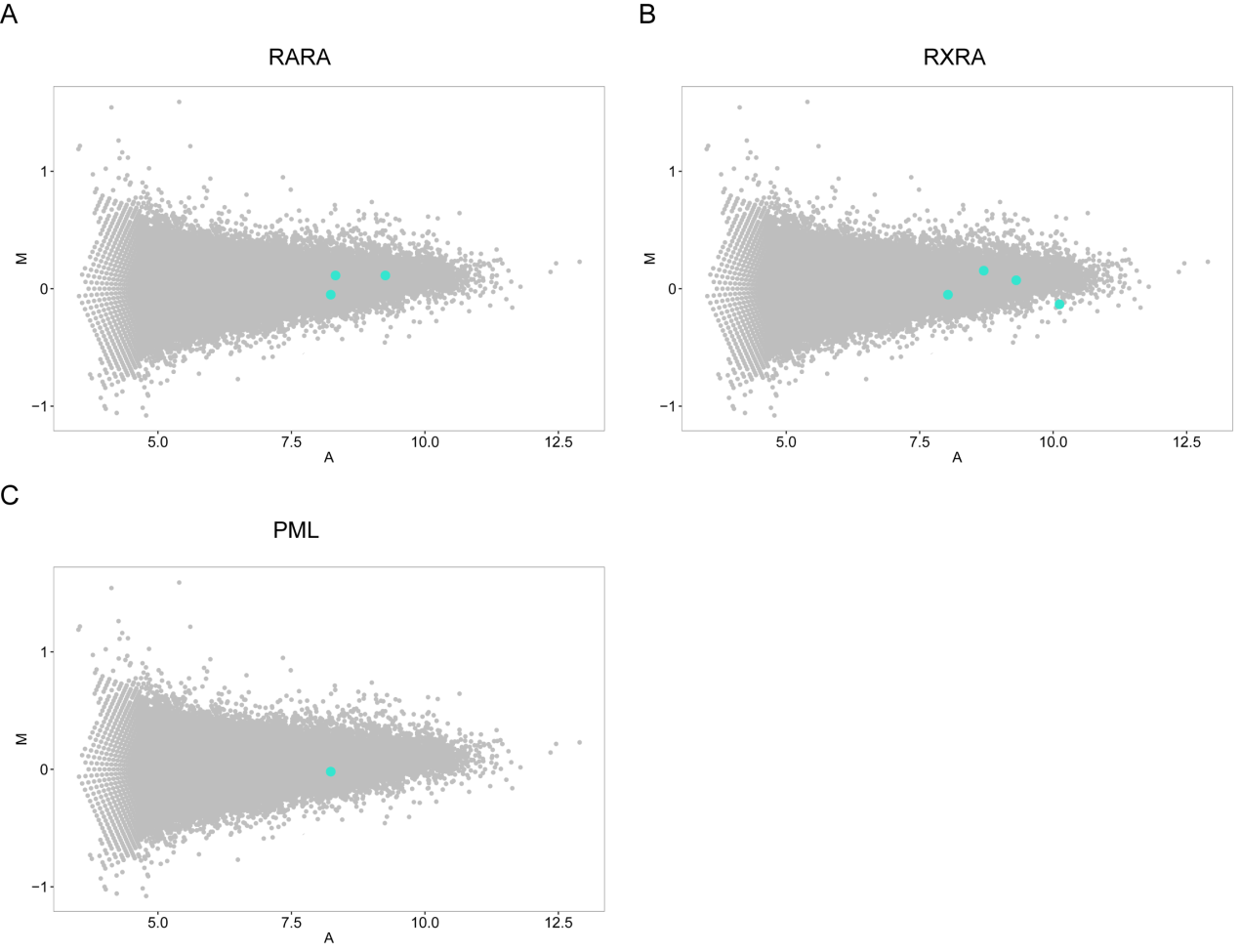


Supplementary Figure S13: MA-plot showing the differential interaction analysis of known retinoic acid signaling related genes: RARA (A), RXRA (B) and PML (C). Specific gene-associated interactions are indicated by the cyan dots.


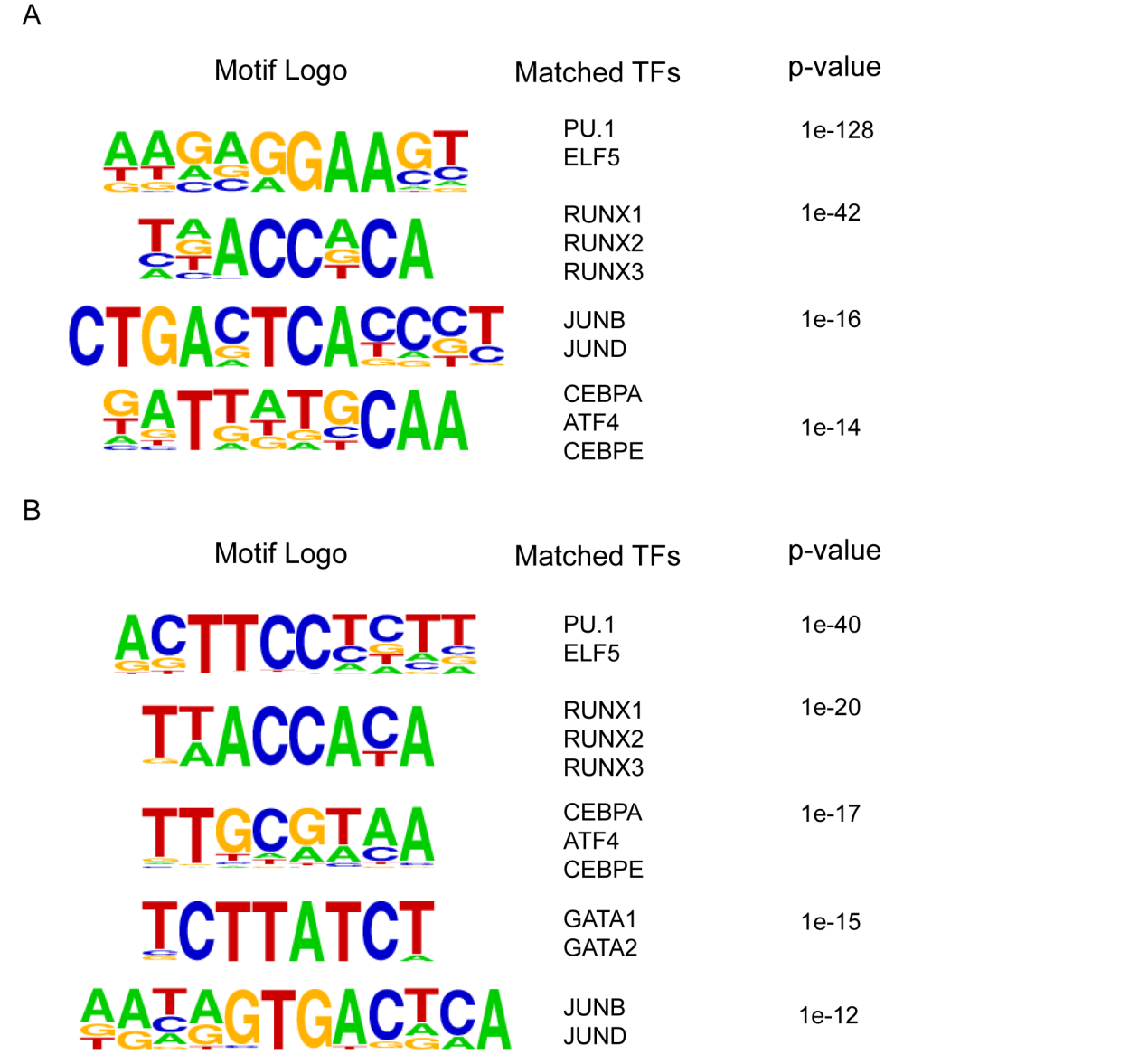


Supplementary Figure S14: Homer de novo motif analysis of ATAC-seq peaks in regulatory regions of Gain (A) or Loss (B) interactions. Motifs are ordered by significance from top to bottom. Compared with the results obtained using control- or ATRA-specific peaks, the CTCF was no longer enriched, and the GATA motif was only significantly enriched in regulatory regions of Loss interactions.


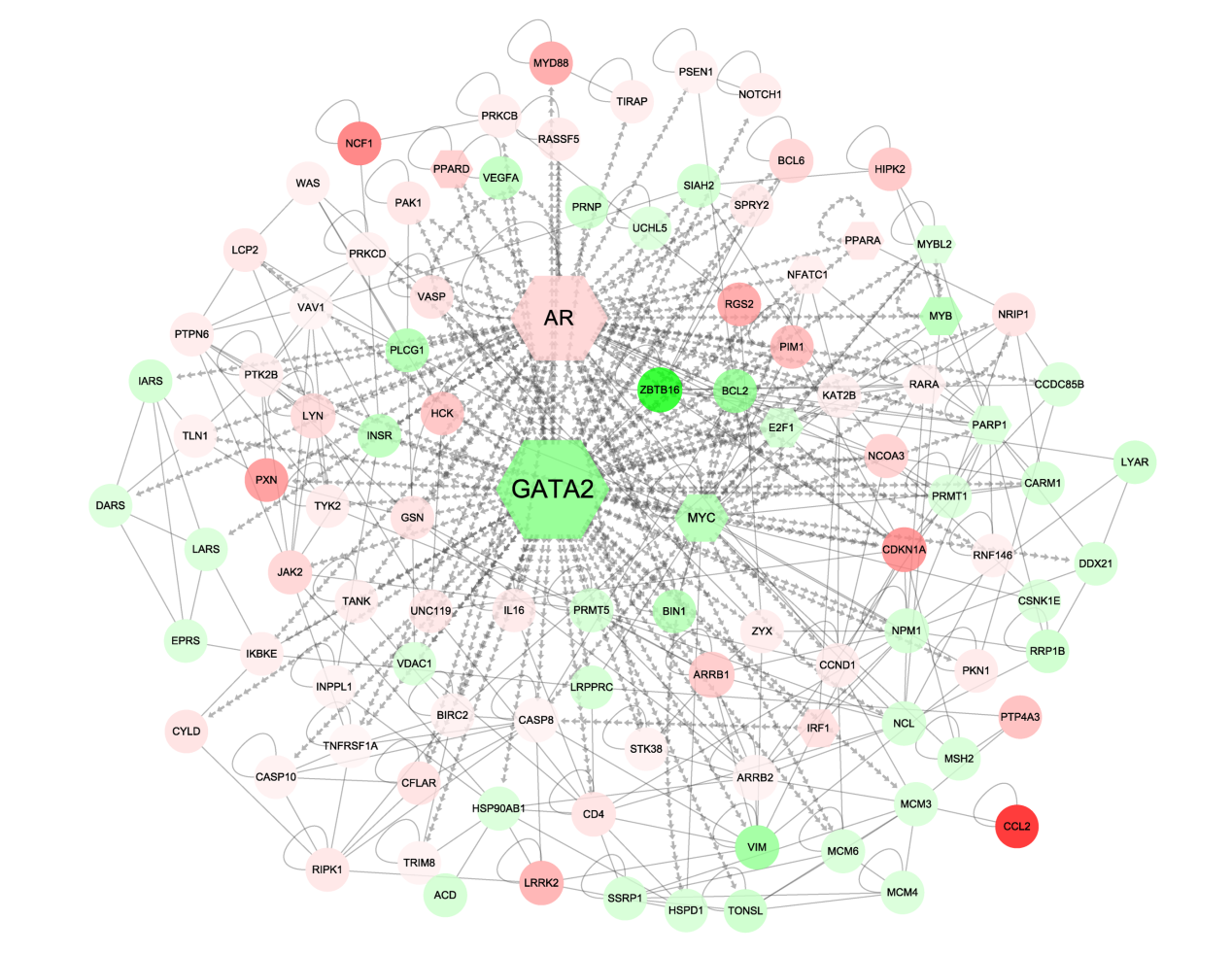


Supplementary Figure S15: Network constructed using differentially expressed genes based on the HTRI database (2). Size of each node represents the linkage betweenness of the node. Color of each node represents the expression fold change of the gene: green indicates down-regulation, and red indicates up-regulation.


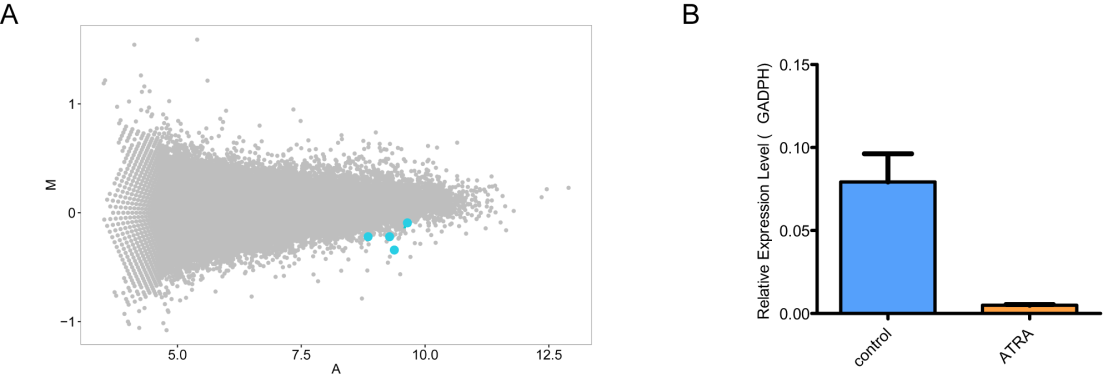


Supplementary Figure S16: (A) MA-plot showing the differential interaction analysis; GATA2-associated interactions are indicated by cyan dots. (B) qPCR result showing significant down-regulation of GATA2 upon ATRA induction.


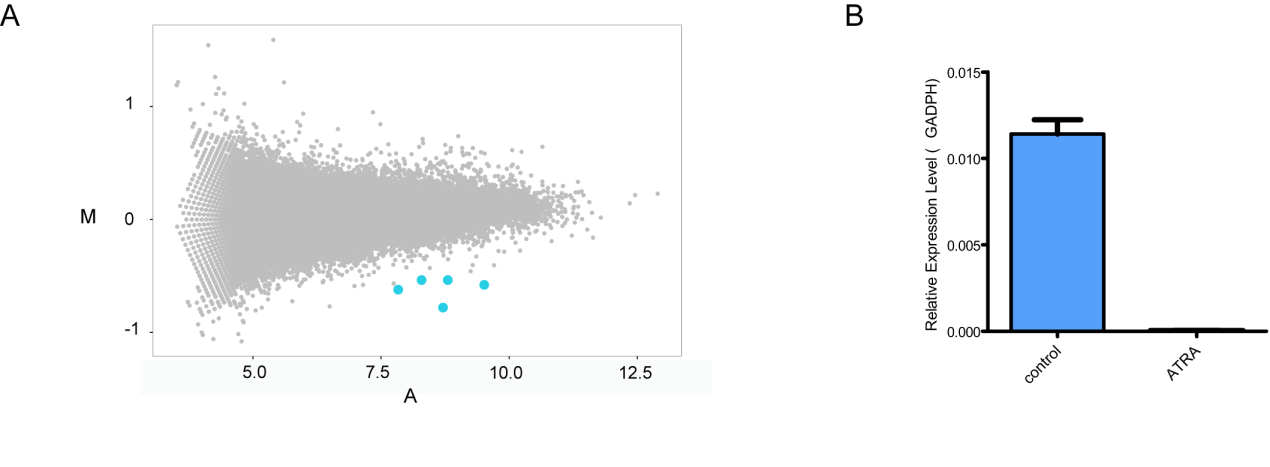


Supplementary Figure S17: Loop between terminator and promoter of ZBTB16 disappeared upon ATRA induction (A) MA-plot showing differential interaction analysis; ZBTB16-associated interactions are indicated by cyan dots. (B) qPCR result showing significant down-regulation of ZBTB16 upon ATRA induction.


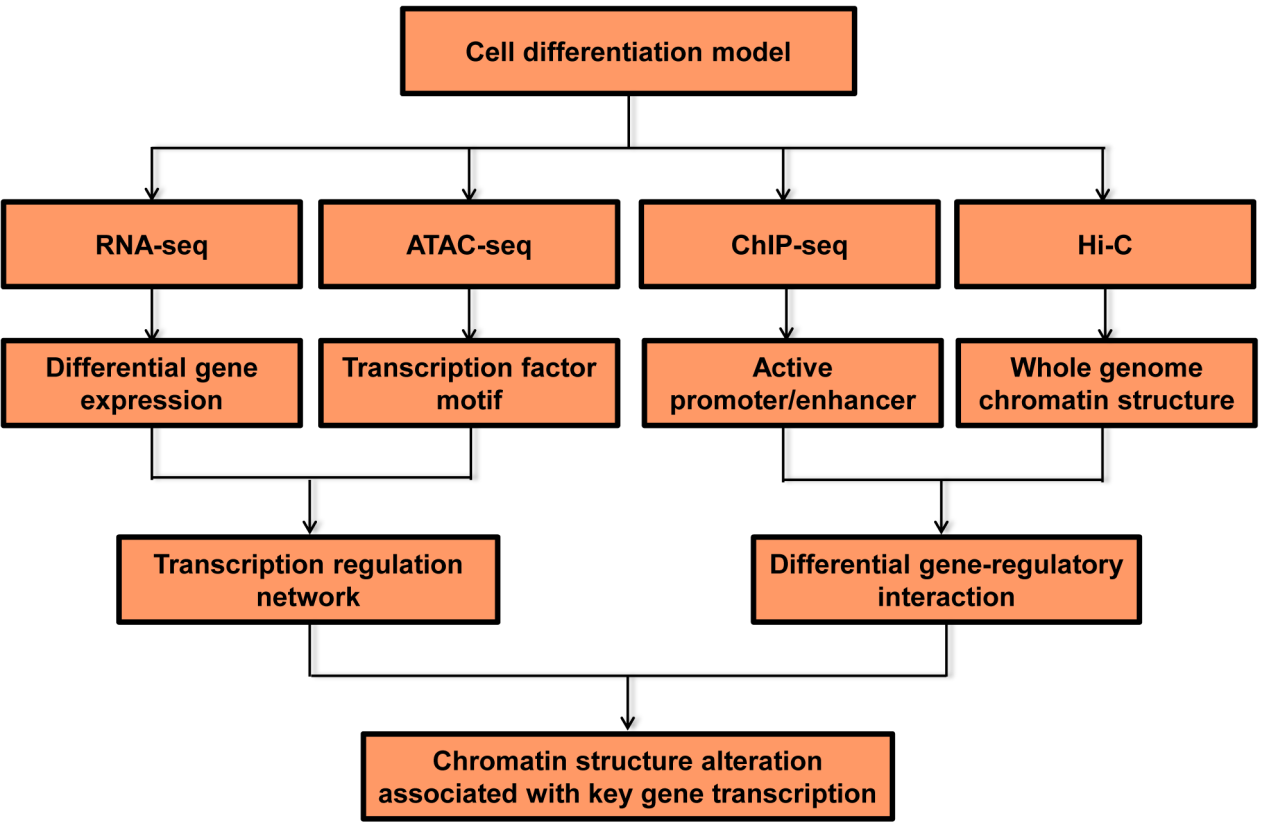


Supplementary Figure S18: Flow diagram showing the framework used to integrate multiple omics data to identify chromatin structure alterations associated with key gene transcription in a cell differentiation model. We named this method RADAR (f**RA**mework of 3**D**genomic rese**AR**ch)

**Reference**

1. Rosenbauer,F. and Tenen,D.G. (2007) Transcription factors in myeloid development: balancing differentiation with transformation. *Nat Rev Immunol*, **7**, 105–117.

2. Bovolenta,L.A., Acencio,M.L. and Lemke,N. (2012) HTRIdb: an open-access database for experimentally verified human transcriptional regulation interactions. *BMC Genomics*, **13**, 405.
